# BAF60c prevents abdominal aortic aneurysm formation through epigenetic control of vascular smooth muscle cell homeostasis

**DOI:** 10.1172/JCI158309

**Published:** 2022-11-01

**Authors:** Guizhen Zhao, Yang Zhao, Haocheng Lu, Ziyi Chang, Hongyu Liu, Huilun Wang, Wenying Liang, Yuhao Liu, Tianqing Zhu, Oren Rom, Yanhong Guo, Lin Chang, Bo Yang, Minerva T. Garcia-Barrio, Jiandie D. Lin, Y. Eugene Chen, Jifeng Zhang

**Affiliations:** 1 Frankel Cardiovascular Center, Department of Internal Medicine, University of Michigan Medical Center, Ann Arbor, Michigan, USA.; 2 Department of Pathology and Translational Pathobiology, Louisiana State University Health Science Center–Shreveport, Shreveport, Louisiana, USA.; 3 Department of Cardiac Surgery, University of Michigan Medical Center, Ann Arbor, Michigan, USA.; 4 Life Sciences Institute and Department of Cell & Developmental Biology, University of Michigan, Ann Arbor, Michigan, USA.

**Keywords:** Vascular Biology, Cardiovascular disease

## Abstract

Abdominal aortic aneurysm (AAA) is a life-threatening vascular disease. BAF60c, a unique subunit of the SWItch/sucrose nonfermentable (SWI/SNF) chromatin remodeling complex, is critical for cardiac and skeletal myogenesis, yet little is known about its function in the vasculature and, specifically, in AAA pathogenesis. Here, we found that BAF60c was downregulated in human and mouse AAA tissues, with primary staining to vascular smooth muscle cells (VSMCs), confirmed by single-cell RNA-sequencing. In vivo studies revealed that VSMC-specific knockout of Baf60c significantly aggravated both angiotensin II– (Ang II–) and elastase-induced AAA formation in mice, with a significant increase in elastin degradation, inflammatory cell infiltration, VSMC phenotypic switch, and apoptosis. In vitro studies showed that BAF60c knockdown in VSMCs resulted in loss of contractile phenotype, increased VSMC inflammation, and apoptosis. Mechanistically, we demonstrated that BAF60c preserved VSMC contractile phenotype by strengthening serum response factor (SRF) association with its coactivator P300 and the SWI/SNF complex and suppressing VSMC inflammation by promoting a repressive chromatin state of NF-κB target genes as well as preventing VSMC apoptosis through transcriptional activation of KLF5-dependent B cell lymphoma 2 (BCL2) expression. Our identification of the essential role of BAF60c in preserving VSMC homeostasis expands its therapeutic potential in preventing and treating AAA.

## Graphical abstract

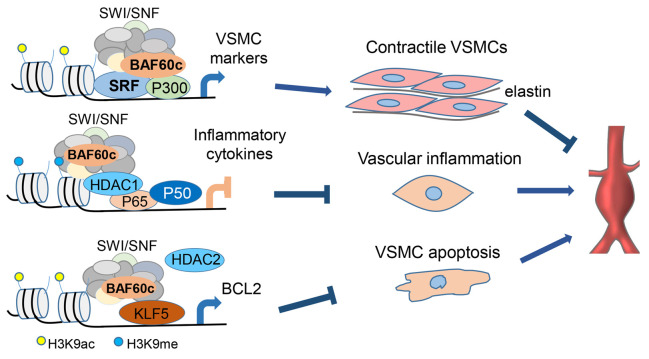

## Introduction

Abdominal aortic aneurysm (AAA) is one of the leading causes of death in developed countries, with an overall mortality rate of approximately 90% in case of rupture (1). AAA usually affects the infrarenal aorta and is typically associated with well-recognized risk factors, including aging, smoking, male sex, hypertension, and atherosclerosis (2). Vascular smooth muscle cells (VSMCs) are critical to maintaining the integrity of healthy vessels, yet retain remarkable plasticity in response to environmental stimuli. Although VSMC dysfunction, including apoptosis and phenotypic switch, have been shown to contribute to AAA development (3–5), the precise control of these processes at the chromatin level remains largely unexplored. Without effective pharmacological therapy available for AAA so far, the current clinical interventions are limited to surgical or endovascular repairs, with only 10% of AAA patients eligible (1, 6). This is likely due to the incomplete understanding of the molecular mechanisms underlying AAA development.

The SWItch/sucrose nonfermentable (SWI/SNF) chromatin-remodeling complex, also known as the BRG1/BRM-associated factor (BAF, where BRM indicates Brahma) complex, interacting with serum response factor (SRF) and myocardin is critical for the transcriptional activation of smooth muscle cell (SMC) contractile genes (7, 8). In the SWI/SNF complex, BAF60 subunits comprise 3 members: BAF60a, BAF60b, and BAF60c (encoded by *SMARCD1*, *SMARCD2*, and *SMARCD3*, respectively) (9). BAF60c, in particular, is required for the development of skeletal and cardiac muscle, acting by orchestrating the interactions between transcription factors and the SWI/SNF complex (9–12). A previous microarray study reported reduced BAF60c expression in human AAA tissues, although its role and cell type–specific expression was not addressed (13). In multipotent adult progenitor cells, BAF60c overexpression activates SMC marker genes in the absence of exogenous cytokines (14). These data suggest an essential role of BAF60c in VSMC differentiation and vascular homeostasis.

In the present study, we show that loss of BAF60c aggravates AAA, indicating a protective role of BAF60c-dependent chromatin remodeling in AAA through preservation of the VSMC contractile phenotype and inhibition of VSMC inflammation and apoptosis. Through systematic analysis of the contributions of BAF60c to AAA pathology and VSMC biology, we provide mechanistic insights into the epigenetic regulation of AAA. This work suggests that local manipulation of BAF60c may constitute a potential therapeutic strategy for AAA.

## Results

### BAF60c is reduced in human and murine AAA.

**Figure 1:**
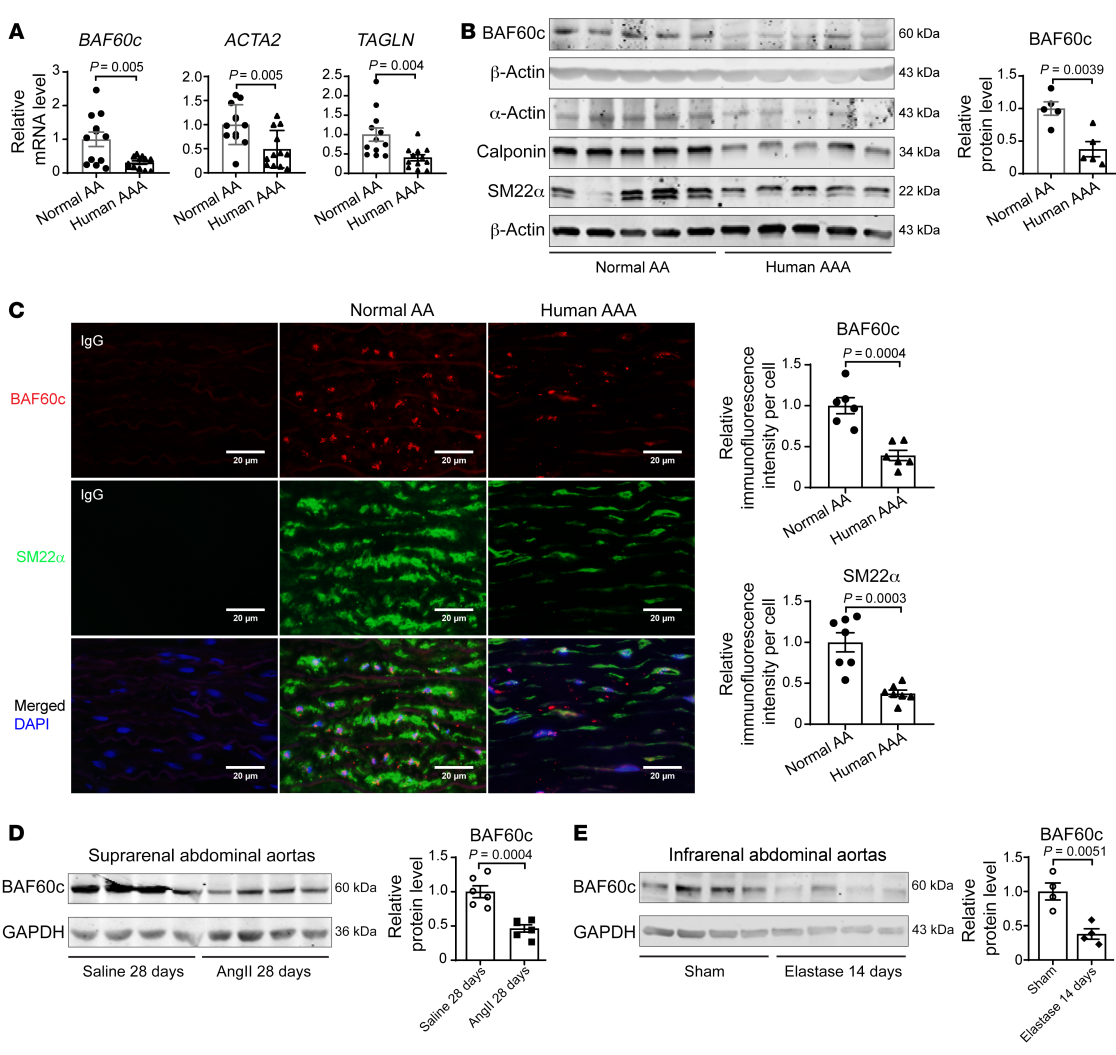
BAF60c is reduced in human and murine AAA tissues. (**A**) *BAF60c*, *ACTA2* (encoding α-actin), and *TAGLN* (encoding SM22α) mRNA levels, relative to GAPDH, were determined by qPCR in human AAA and normal abdominal aortas (AA) (*n* = 12/group). (**B**) Protein abundance of BAF60c, SM α-actin, calponin, and SM22α were determined by Western blot in human AAA samples and normal abdominal aortas (*n* = 5/group). (**C**) Representative immunofluorescence staining and quantification of BAF60c (red) and SM22α (green) in human AAA and normal abdominal aortas (*n* = 6/group). Nuclei stained with DAPI are blue. Scale bars: 20 μm. (**D**) BAF60c in the suprarenal abdominal aortas of C57BL/6J mice injected intraperitoneally with AAV-*Pcsk9.D377Y* and infused with saline or Ang II (1000 ng/kg/min) for 28 days was determined by Western blot (*n* = 6/group). (**E**) BAF60c in the infrarenal abdominal aortas of C57BL/6J mice was determined by Western blot 14 days after elastase exposure or heat-inactivated elastase exposure for 30 minutes (*n* = 4/group). Data are represented as mean ± SEM. Student’s *t* test (**A**–**E**).

To explore the role of BAF60c in AAA development and VSMC phenotypic modulation, we first assessed the expression of BAF60c and VSMC contractile markers in human AAA samples and normal aortic tissues (Supplemental Table 1; supplemental material available online with this article; https://doi.org/10.1172/JCI158309DS1). BAF60c was significantly decreased in the aneurysmal tissue; this was accompanied by reduced VSMC contractile markers, including smooth muscle (SM) α-actin, calponin, and SM22α (Figure 1, A and B), which was further confirmed by immunofluorescence staining (Figure 1C). Similarly, BAF60c and SM22α were also significantly decreased in murine AAA tissues from *Pcsk9*/angiotensin II– (Ang II– ) (Figure 1D and Supplemental Figure 1, A and B) and elastase-induced AAA models (Figure 1E and Supplemental Figure 1C). Reanalysis of our single-cell RNA-sequencing (scRNA-Seq) data set of infrarenal abdominal aortas (15) showed that BAF60c is selectively expressed in SMCs (Supplemental Figure 1D). Collectively, these data indicate that BAF60c may play an essential role in AAA.

### BAF60c depletion in VSMCs aggravates AAA development in mice.

**Figure 2:**
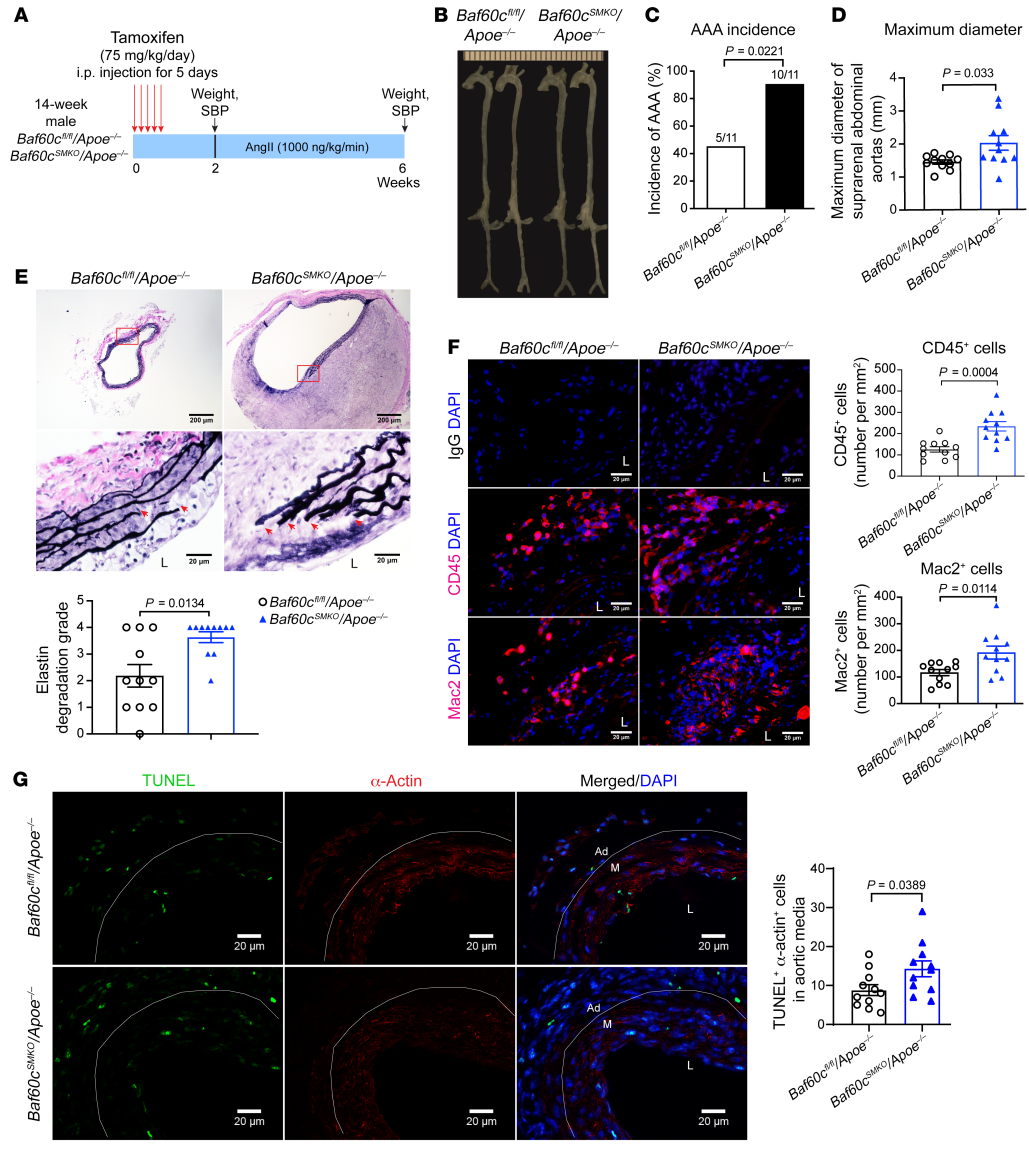
VSMC-BAF60c deficiency aggravates Ang II–induced AAA in mice. (**A**–**H**) Sixteen-week-old male *Baf60c^fl/fl^* *Apoe^–/–^* and *Baf60c*^SMKO^ *Apoe^–/–^* mice (*n* = 11/group) were infused with Ang II (1000 ng/kg/min) for 4 weeks. (**A**) Schematic of Ang II–induced AAA model. (**B**) Representative morphology of aortas at the end point. (**C**) AAA incidence (*n* = 11/group). (**D**) Quantification of maximum external diameters of suprarenal abdominal aortas (AA) (*n* = 11/group). (**E**) Representative images of VVG staining and analysis of elastin fragmentation in the sections of suprarenal abdominal aortas (*n* = 11/group). Scale bars: 200 μm (whole sections); 20 μm (higher magnification areas). L, lumen. (**F**) Representative immunofluorescence staining and quantification of leukocyte (CD45^+^, red) and macrophage (Mac2^+^, red) infiltration in the aortic wall of suprarenal abdominal aortas. Scale bars: 20 μm. (**G**) Representative TUNEL (green) and α-actin (red) staining and quantification of the apoptotic α-actin–positive cells in the media of suprarenal abdominal aortas (*n* = 11/group). Scale bars: 20 μm. Ad, adventitia. M, media. Nuclei stained with DAPI are blue. Data are represented as mean ± SEM. χ^2^ Test (**C**); Student’s *t* test (**D**, **F**, and **G**); Mann-Whitney *U* test (**E**).

VSMCs are essential for vascular homeostasis, and their dysfunction contributes to AAA (3, 16). To evaluate the role of BAF60c in VSMCs in AAA development, we generated VSMC-specific *Baf60c*-KO mice (*Baf60c*^SMKO^) by crossbreeding *Baf60c* floxed mice (*Baf60c^fl/fl^*) with *Myh11*-CreER^T2^ mice; this was followed by tamoxifen induction (Supplemental Figure 2A). The *Baf60c* KO in VSMCs was confirmed by quantitative PCR (qPCR) and Western blotting (Supplemental Figure 2, B and C). After crossbreeding with hyperlipidemic *Apoe^–/–^* and tamoxifen induction, *Baf60c*^SMKO^ *Apoe^–/–^* and *Baf60c^fl/fl^* *Apoe^–/–^* mice were used for the Ang II–induced AAA model (Figure 2A) (17). After 4 weeks of Ang II (1,000 ng/kg/min) infusion, body weight, blood pressure, and plasma lipid profiles were comparable between the 2 groups (Supplemental Figure 2, D–F). Nevertheless, VSMC-specific Baf60c (VSMC-*Baf60c*) deficiency significantly increased AAA incidence (90.9% vs. 45.5%) and maximum aortic diameters (2.017 ± 0.222 mm vs. 1.447 ± 0.064 mm) (Figure 2, B–D). Concomitantly, elastin degradation and leukocyte (CD45^+^) and macrophage (Mac2^+^) accumulation in the aortic wall and the concentration of plasma MCP-1 and IL-6 were significantly increased in *Baf60c*^SMKO^ *Apoe^–/–^* mice (Figure 2, E and F, and Supplemental Figure 2G). VSMC transition to a macrophage-like state or apoptosis both contribute to AAA development (3–5, 18). VSMC-*Baf60c* KO increased phenotypically modulated VSMCs costained by SM22α and Mac2, and VSMC apoptosis was assessed by TUNEL and α-actin costaining (Supplemental Figure 2H and Figure 2G).

The protective role of VSMC-*Baf60c* in AAA was further confirmed in the elastase-induced AAA model. Fourteen days after elastase exposure, there was no noticeable difference in body weight, blood pressure, or plasma lipid profiles as well as the concentration of MCP-1 and IL-6 between the 2 groups (Supplemental Figure 3, A–E). Consistently, *Baf60c*^SMKO^ mice exhibited increased aortic enlargement, elastin degradation, and leukocyte and macrophage accumulation as well as increased phenotypically modulated VSMCs and apoptotic VSMCs in the aortic wall (Supplemental Figure 3, F–K). Collectively, the above findings indicate that the loss of VSMC-BAF60c promotes AAA development.

To explore the therapeutic potential of BAF60c for AAA in vivo, an adenovirus carrying the human *BAF60c* gene (Ad-*BAF60c*, 2 × 10^9^ viral particles/mouse) was delivered periadventitially to the suprarenal abdominal aorta of C57BL/6J mice after the Pcsk9/Ang II AAA model had been induced for 2 weeks (Supplemental Figure 4A). BAF60c overexpression did not improve the survival rate (Supplemental Figure 4B). The diameter of the suprarenal abdominal aorta was monitored by ultrasound imaging before and after adenovirus delivery (Supplemental Figure 4, C–E). BAF60c overexpression significantly reduced the maximum diameters of the suprarenal abdominal aorta and AAA incidence (Supplemental Figure 4, E–H).

### BAF60c contributes to preserving the VSMC contractile phenotype.

**Figure 3:**
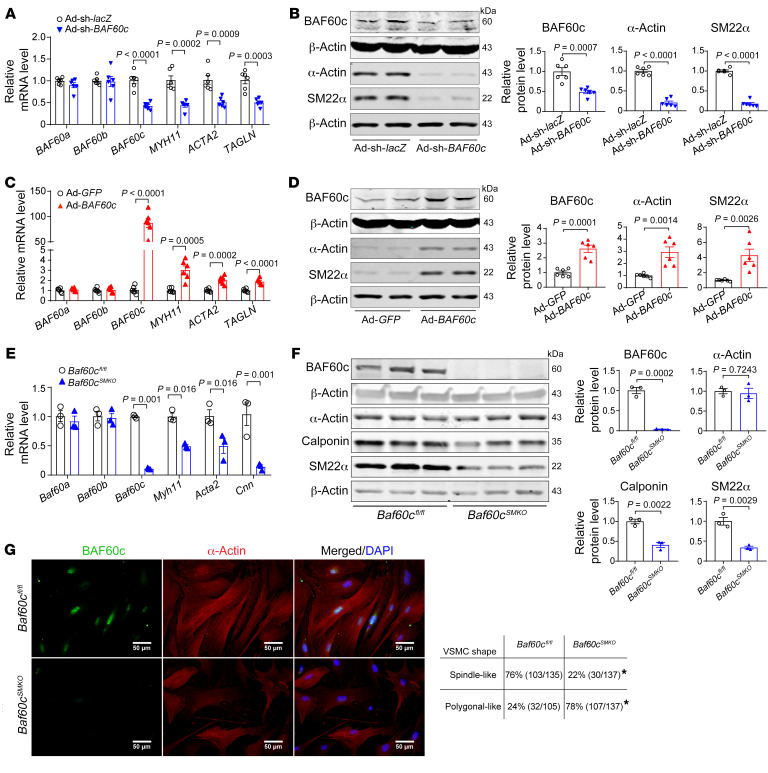
BAF60c preserves VSMC contractile phenotype. (**A**–**D**) HASMCs were infected with Ad-sh-*lacZ* and Ad-sh-*BAF60c* (10 MOI) (**A** and **B**) or Ad-*GFP* and Ad-*BAF60c* (10 MOI) (**C** and **D**). After 48 hours, cells were serum starved in Opti-MEM for 24 hours. mRNA levels of *BAF60c*, *ACTA2*, and *CNN1* (**A** and **C**) and protein abundance of BAF60c, α-actin, and SM22α (**B** and **D**) were determined from 6 independent experiments. (**E**–**G**) MASMCs were isolated from *Baf60c^fl/fl^* and *Baf60c*^SMKO^ mice 9 days after 5 consecutive days of tamoxifen (75 mg/kg/d) intraperitoneal injection. qPCR (**E**) and Western blot (**F**) to determine the expression of Baf60c, Myh11, α-actin, calponin, and SM22α in MASMCs. (**G**) Representative immunofluorescence images of BAF60c and α-actin staining of MASMCs and quantification of the percentage of spindle-like or polygonal-like cells. Nuclei stained with DAPI are blue. Scale bars: 50 μm. Data are represented as mean ± SEM. Student’s *t* test for (**A**–**F**); χ^2^ test (**G**). *P < 0.05.

Our scRNA-Seq data showed that BAF60c is primarily expressed in SMCs (Supplemental Figure 1D). Additionally, BAF60c and VSMC contractile genes were decreased in synthetic VSMCs induced by PDGF-BB (20 ng/mL), but upregulated in TGF-β–treated VSMCs (10 ng/mL) (Supplemental Figure 5, A and B). Next, we found that knockdown of *BAF60c* with shRNA significantly decreased the expression of VSMC contractile markers in human aortic SMCs (HASMCs) (Figure 3, A and B). Conversely, adenovirus-mediated *BAF60c* overexpression significantly upregulated those contractile markers (Figure 3, C and D). These results were further validated in A7r5 cells and mouse aortic SMCs (MASMCs) isolated from *Baf60c*^SMKO^ and control mice (Supplemental Figure 5, C and D, and Figure 3, E and F). Accordingly, *Baf60c* deficiency in MASMCs or knockdown in serum-starved A7r5 cells facilitated VSMC phenotypic switch from an elongated contractile phenotype to a polygonal synthetic phenotype (Figure 3G and Supplemental Figure 5E). Notably, *BAF60c* deficiency or overexpression did not affect the expression of *BAF60a* and *BAF60b* (Figure 3, A, C, and E). These data suggest that BAF60c is required to maintain the VSMC contractile phenotype.

### BAF60c acts as a bridge between SRF and the SWI/SNF chromatin remodeler.

**Figure 4:**
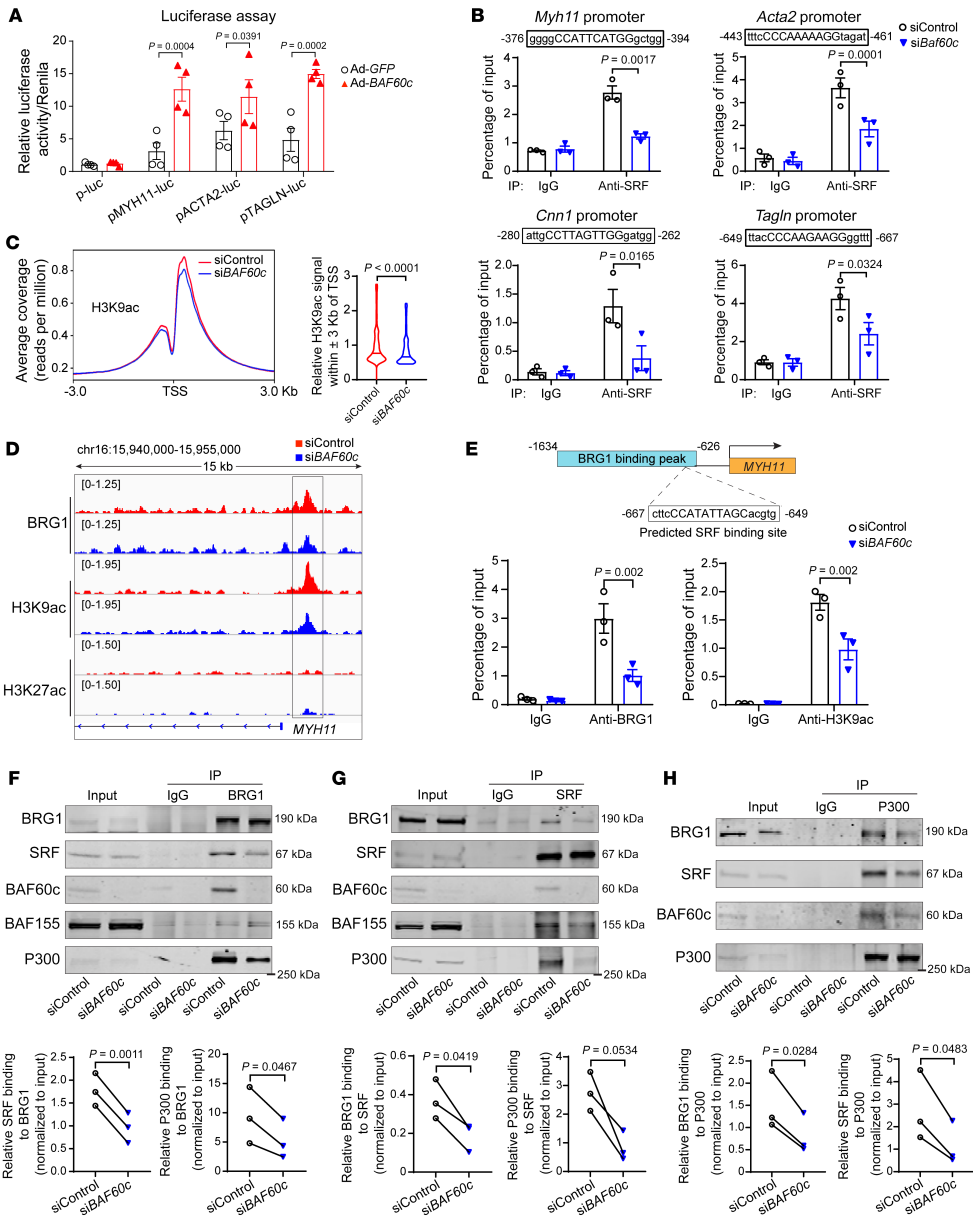
BAF60c acts as a bridge between SRF and the SWI/SNF complex. (**A**) Luciferase assays in A7r5 cells transfected with *MYH11*, *ACTA2*, or *TAGLN* promoter-driven luciferase reporters and infected with Ad-*GFP* or Ad-*BAF60c* (10 MOI). After 48 hours, the promoter activities were determined by dual luciferase assay and normalized by Renilla activity (*n* = 4). (**B**) A7r5 cells were transfected with siControl or si*Baf60c* (30 nM). After 48 hours, cells were serum starved in Opti-MEM for 24 hours. ChIP assays were performed to determine SRF-binding activity to *Myh11*, *Acta2*, *Cnn1*, and *Tagln* promoters. IgG served as control. (**C** and **D**) HASMCs were transfected with siControl or si*BAF60c* (30 nM). After 48 hours, cells were serum starved in Opti-MEM for 24 hours and ChIP-Seq was performed using BRG1, H3K9ac, and H3K27ac Abs. (**C**) Histogram of ChIP-Seq reads of H3K9ac ±3 Kb surrounding the TSS of genes (left). Quantification of relative read ratio of the H3K9ac signals within the TSS ± 3 Kb is presented in box plots (right). (**D**) IGV image showing localization of BRG1 and H3K9ac and H3K27ac within *MYH11* promoter in HASMCs. (**E**) BRG1 and H3K9ac binding on the predicted SRF-binding site located in the human *MYH11* promoter were determined by ChIP assay in HASMCs transfected with siControl or si*BAF60c* (30 nM) for 48 hours, followed by serum starvation in Opti-MEM for 24 hours. (**F**–**H**) HASMCs were transfected with siControl or si*BAF60c* (30 nM). After 48 hours, cells were serum starved in Opti-MEM for another 24 hours, and nuclear proteins were isolated and subjected to Co-IP using BRG1, SRF, or P300 Abs. IgG was the negative control. Three independent experiments were performed for **B** and **E**–**H**. Data are represented as mean ± SEM. Two-way ANOVA followed by Holm-Šidák post hoc analysis (**A**, **B**, and **D**); Mann-Whitney *U* test (**C**); paired *t* test (**F**–**H**).

SRF binding to the CArG box (CC[A/T]_6_GG) within the promoters of nearly all VSMC contractile genes is required for transcriptional activation of those genes (19). Our results revealed that BAF60c overexpression in A7r5 cells increased the activity of *MYH11*, *ACTA2*, and *TAGLN* promoter-driven luciferase reporters (20, 21) (Figure 4A). Next, we performed ChIP assays and found that *Baf60c* knockdown diminished SRF binding to the predicted CArG boxes within the promoters of *Myh11* (–375/–394 bp), *Acta2* (–443/–461 bp), *Cnn1* (–280/–262 bp), and *Tagln* (–649/–667bp) (Figure 4B) without altering the expression of BRG1, SRF, and myocardin (Supplemental Figure 6A). Histone acetylation also accompanies SRF binding to the CArG box in the regulation of SMC gene expression (19). To further determine whether BAF60c-dependent transcriptional activation of SRF target genes is associated with chromatin accessibility and histone modifications within gene promoters, we performed ChIP-Seq after chromatin pull-down with Abs against BRG1 or acetyl-histone H3 (Lys9) (H3K9ac) and acetyl-histone H3 (Lys27) (H3k27ac), 2 transcriptional activation markers (22), in HASMCs transfected with either nontargeting control siRNA (siControl) or si*BAF60c*. *BAF60c* knockdown caused a decrease in the H3K9ac signal in proximity to the transcription start site (TSS) (Figure 4C) without altering the expression of histone acetyltransferase (P300) and histone deacetylase (HDAC2) (Supplemental Figure 6A), indicating reduced chromatin accessibility at those promoters upon *BAF60c* knockdown. Through ChIP-Seq, we found diminished BRG1 binding at the *MYH11* promoter, accompanied by a decreased enrichment of H3K9ac near the BRG1-binding region (Figure 4D). Furthermore, the ChIP assay upon *BAF60c* knockdown confirmed the reduced BRG1 binding and H3K9ac signal around the predicted SRF-binding site (–667/–649 bp), which resides within the BRG1-binding region (–1634/–626 bp) (Figure 4E). BAF60c was demonstrated to interact with myocardin, a coactivator of SRF, to regulate target genes essential for heart development (10). Next, we performed coimmunoprecipitation (co-IP) and found an interaction between the SWI/SNF complex containing BAF60c and SRF in contractile VSMCs (Supplemental Figure 6B). In addition, *BAF60c* knockdown in serum-starved HASMCs significantly reduced the interaction between the SWI/SNF complex and SRF (Figure 4, F and G). SRF interaction with coactivators, such as P300, and subsequent recruitment of chromatin-remodeling machinery, such as the SWI/SNF complex, is critical for transcriptional activation of VSMC-specific genes (7, 8). Consistently, P300 association with the SWI/SNF complex and SRF was significantly reduced upon *BAF60c* knockdown in HASMCs (Figure 4H). Of note, *BAF60c* overexpression did not alter the interaction of the SWI/SNF complex with SRF or P300, but increased SRF-P300 interaction (Supplemental Figure 6C). Collectively, these data suggest that BAF60c is essential for the interaction of the SWI/SNF complex with SRF and for the epigenetic control of SRF binding to the CArG element in VSMC marker genes, thereby preserving the VSMC contractile phenotype.

### BAF60c negatively regulates VSMC inflammation.

**Figure 5:**
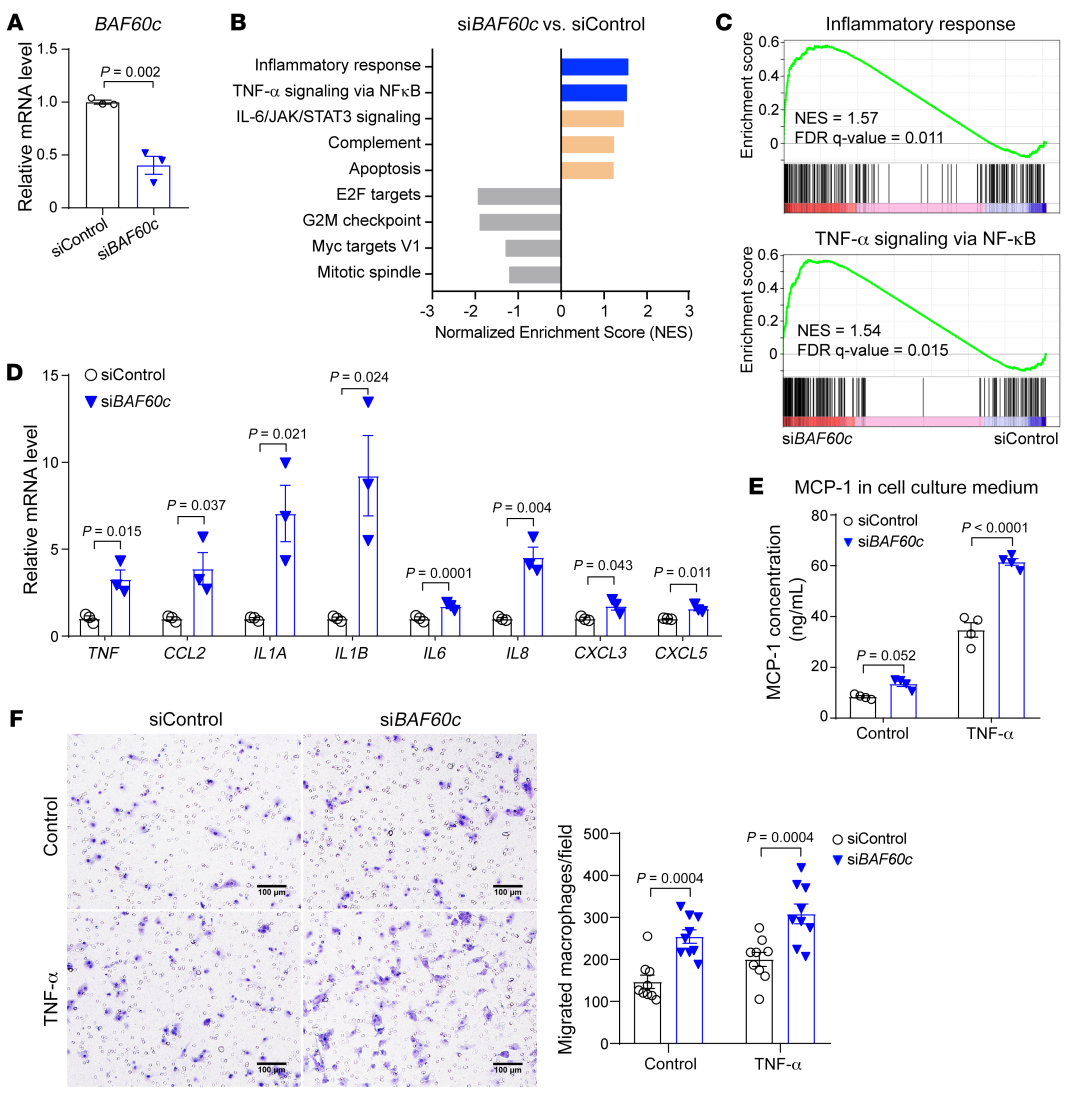
BAF60c depletion enhances VSMC inflammatory response. (**A**–**C**) HASMCs were transfected with siControl or si*BAF60c* (30 nM). After 24 hours, cells were serum starved in Opti-MEM for 24 hours, followed by total RNA extraction for qPCR (**A**) and for RNA-Seq (**B** and **C**). (**B**) GSEA was performed comparing si*BAF60c* with siControl. (**C**) Positive enrichment of the inflammatory response (left) and TNF-α signaling via NF-κB (right) in GSEA plots (si*BAF60c* vs. siControl). (**D**) qPCR to validate the expression of inflammation-related genes in 3 independent sets of samples treated as in **A**. (**E**) MCP-1 concentration was measured by ELISA in cell culture media. HASMCs were transfected with siControl or si*BAF60c*. After 48 hours, cells were cultured in opti-MEM with or without TNF-α (20 ng/mL) for 24 hours. Data are from 4 independent experiments. (**F**) Representative images (magnified field, left) and quantitative analysis (right) of BMDMs (isolated from WT mice) in the Transwell migration assay cocultured with HASMCs transfected with siControl or si*BAF60c* in the presence or absence of TNF-α (20 ng/mL) (*n* = 9 images/group). Scale bars: 100 μm. Data are represented as mean ± SEM. Student’s *t* test (**A** and **D**); 2-way ANOVA followed by Holm-Šidák post hoc analysis (**E** and **F**).

To further explore the molecular mechanisms underlying the role of BAF60c in AAA, RNA-Seq was performed to uncover the transcriptomic changes in HASMCs transfected with si*BAF60c* relative to siControl (Figure 5A). Gene Set Enrichment Analysis (GSEA) (23) was used to search for the overrepresented pathways across the Molecular Signatures Database (MSigDB) (Supplemental Table 2). Of note, the inflammatory response and TNF-α signaling via NF-κB were enriched in the upregulated pathways in HASMCs upon *BAF60c* knockdown (Figure 5, B and C, Supplemental Table 3). Next, we validated the upregulation of several proinflammatory cytokines regulated by NF-κB upon *BAF60c* knockdown by qPCR in HASMCs (Figure 5D). Conversely, BAF60c overexpression significantly diminished the expression of those proinflammatory cytokines in HASMCs under TNF-α stimulation (Supplemental Figure 7, A and B). Additionally, *BAF60c* knockdown in HASMCs increased TNF-α–induced MCP-1 secretion and promoted migration of bone marrow–derived macrophages (BMDMs) toward HASMCs (Figure 5, E and F), while *BAF60c* overexpression showed the opposite effect (Supplemental Figure 7, C and D). Taken together, these data suggest that BAF60c inhibits VSMC inflammatory response.

### BAF60c is required for the establishment of repressive chromatin status at the NF-κB target genes.

**Figure 6:**
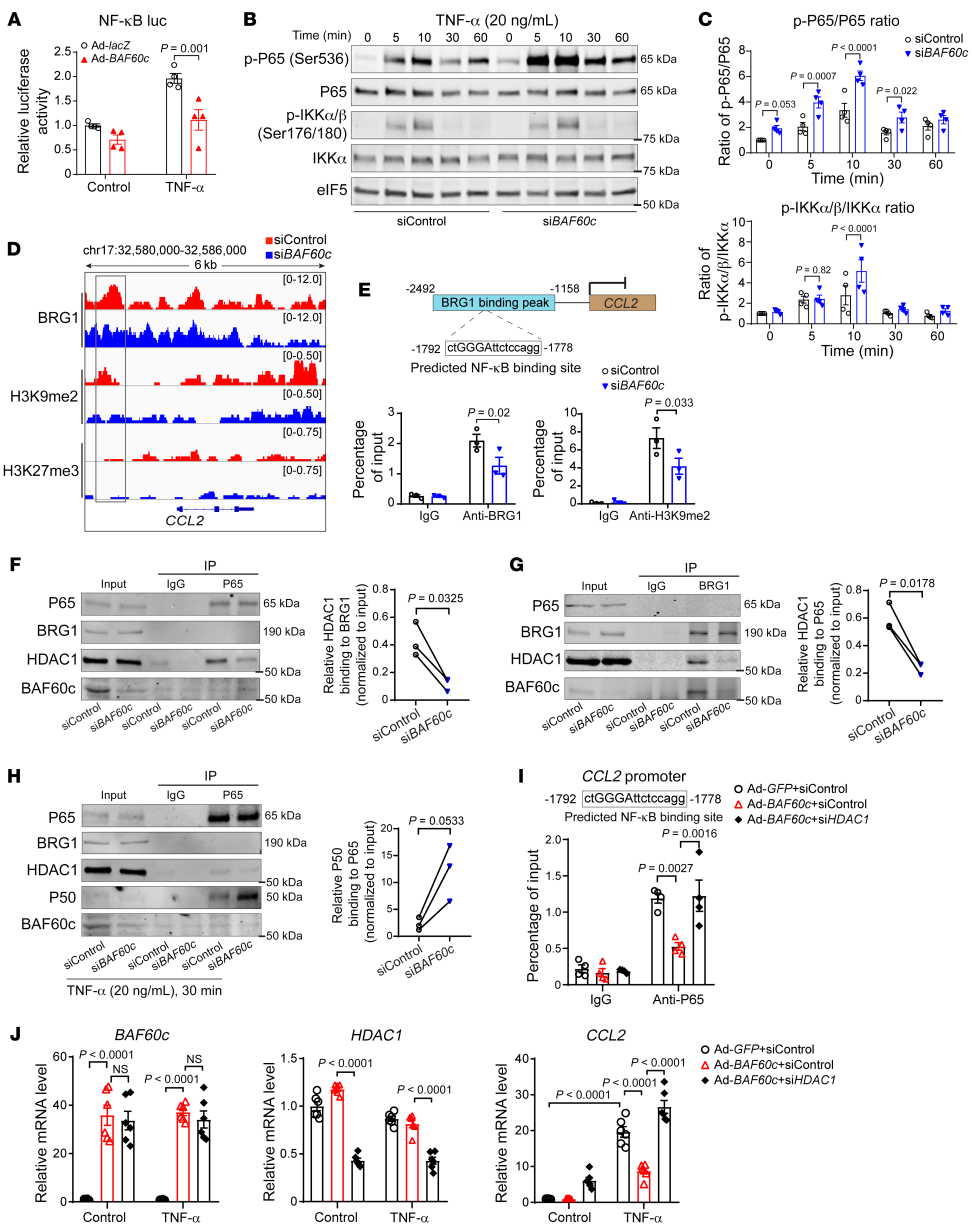
BAF60c knockdown activates the NF-κB pathway. (**A**) HASMCs were transfected with the *NF-κB* response element–derived luciferase reporter. After 8 hours, cells were infected with Ad-*lacZ* or Ad-*BAF60c* (10MOI). After 48 hours, cells were stimulated with TNF-α (20 ng/mL) for 12 hours and promoter activity was determined by dual-luciferase assay and normalized against Renilla luciferase activity. (**B** and **C**) HASMCs were transfected with siControl or si*BAF60c* (30 nM). After 48 hours, cells were serum starved in Opti-MEM for 24 hours and then treated with TNF-α (20 ng/mL). (**B**) Western blot to determine the protein abundance of phosphorylated P65 (p-P65), P65, p-IKKα/β, and IKKα. (**C**) Quantitative analysis of the ratio of p-P65 to P65 and p-IKKα/β to IKKα in **B** (*n* = 3). (**D**) HASMCs were transfected with siControl or si*BAF60c* (30 nM). After 48 hours, cells were serum starved in Opti-MEM for 24 hours, and then ChIP-Seq was performed with Abs against BRG1, H3K9me2, and H3K27me3. Normalized ChIP-Seq reads of BRG1, H3K9me2, and H3K27me3 in human *CCL2* gene promoter and coding region are shown in IGV image. (**E**) BRG1 and H3K9me2 binding at the predicted NF-κB–binding site within the *CCL2* gene promoter were determined by ChIP assay in HASMCs transfected with siControl or si*BAF60c* (30 nM) for 48 hours, serum starved for 24 hours, and then treated with TNF-α (20 ng/mL) for 30 minutes. (**F**, **G**, and **H**) HASMCs were transfected with siControl or si*BAF60c* (30 nM). After 48 hours, cells were serum starved in Opti-MEM for 24 hours and treated with or without TNF-α (20 ng/mL) for 30 minutes, followed by nuclear protein isolation and Co-IP using Abs against P65 or BRG1. IgG served as negative control. (**I** and **J**) HASMCs were transfected with siControl or si*HDAC1* (30 nM) and infected with Ad-*GFP* or Ad-*BAF60c* for 48 hours, serum starved for 24 hours, and then treated with TNF-α (20 ng/mL) for 30 minutes (**I**) or 24 hours (**J**). (**I**) P65 binding at the predicted NF-κB–binding site within the human *CCL2* promoter was determined by ChIP assay. Data are from 4 independent experiments. (**J**) qPCR to determine mRNA levels of *BAF60c*, *HDAC1*, and *CCL2*. Three independent experiments were performed for **E**–**G**. Data are represented as mean ± SEM. Two-way ANOVA followed by Holm-Šidák post hoc analysis (**A**, **C**, **E**, **I**, and **J**); paired *t* test (**F**–**H**).

The NF-κB luciferase reporter assay demonstrated that *BAF60c* overexpression inhibits NF-κB binding to the consensus NF-κB–binding elements (Figure 6A), indicating that BAF60c might negatively regulate the NF-κB pathway. The phosphorylation of IκB and P65/P50 is required to activate the expression of proinflammatory cytokines (24). Accordingly, TNF-α stimulation of HASMCs increased the phosphorylation of IKKα/β and P65 (Figure 6, B and C). Notably, *BAF60c* knockdown in HASMCs significantly increased P65 phosphorylation in the absence or presence of TNF-α without altering IKKα/β phosphorylation (Figure 6, B and C). Histone methylation at gene promoters, particularly H3K9me2 and H3k27me3, contributes to the basal repressive regulation of NF-κB target genes (25, 26). ChIP-Seq was performed for BRG1, H3K9me2, and H3K27me3 in HASMCs. Accordingly, we found reduced BRG1- and H3K9me2-binding peaks at the NF-κB target gene *CCL2* promoter upon *BAF60c* knockdown in HASMCs (Figure 6D). Additionally, an NF-κB–binding site was found within the BRG1-binding peak (Figure 6E). Next, ChIP assays confirmed reduced BRG1 binding and H3K9me2 signal in this NF-κB–binding site within the *CCL2* promoter in HASMC *BAF60c* knockdown (Figure 6E), suggesting that BAF60c is required for the establishment of repressive chromatin status at NF-κB target genes. NF-κB association with corepressors, such as HDAC1, establishes a repressive chromatin state to silence NF-κB target genes in the resting cells (25). Although no association between BRG1 and P65 assessed by co-IP was observed in either TNF-α–treated or untreated VSMCs, P65-HDAC1 interaction was significantly reduced upon *BAF60c* knockdown (Figure 6, F and G). Additionally, knockdown of *BAF60c* enhanced P65 association with its coactivator P50 in TNF-α–treated VSMCs (Figure 6H). In addition, HDAC1 knockdown can abolish the inhibitory effect of BAF60c on the NF-κB pathway and target gene expression in TNF-α–treated VSMCs (Figure 6, I and J). Collectively, these findings suggest that BAF60c-dependent suppression of VSMC inflammation relies on the precise control of P65 association with its repressor HDAC1 and coactivator P50.

### BAF60c inhibits VSMC apoptosis.

**Figure 7:**
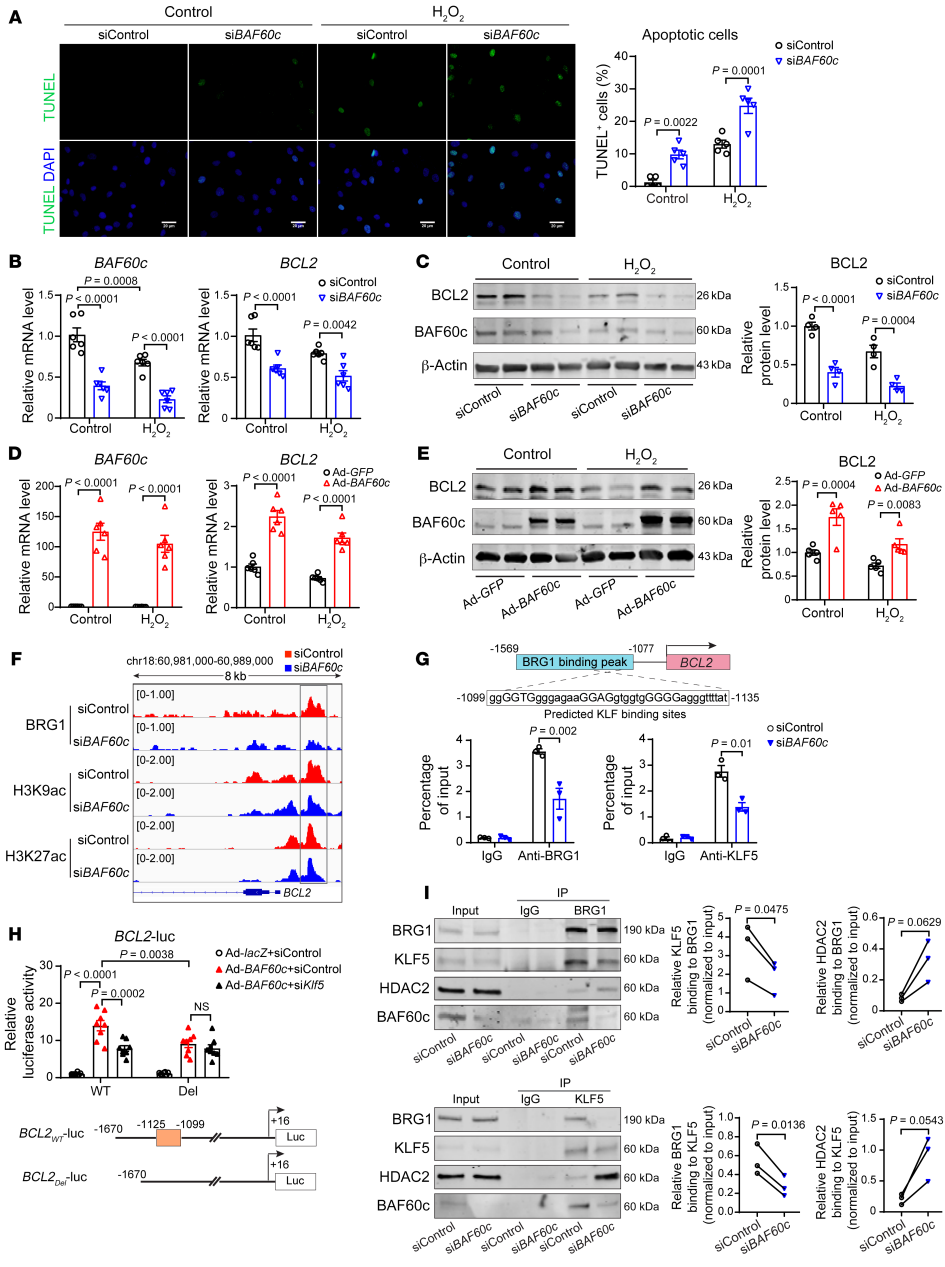
BAF60c inhibits VSMC apoptosis through upregulation of BCL2. (**A**) Representative images of TUNEL staining (green) and quantification of apoptotic HASMCs transfected with siControl or si*BAF60c* (30 nM) for 48 hours and subsequently stimulated for 24 hours with 200 μM hydrogen peroxide (H_2_O_2_). Scale bars: 20 μm. (**B**–**E**) HASMCs were transfected with siControl and si*BAF60c* (30 nM) (**B** and **C**) or infected with Ad-*GFP* and Ad-*BAF60c* (10MOI) (**D** and **E**). After 48 hours, cells were stimulated with 200 μM H_2_O_2_ for 24 hours. Expression of BAF60c and BCL2 was assessed by qPCR (**B** and **D**, *n* = 6) and Western blot (**C** and **E**, *n* = 4). (**F**) HASMCs were transfected with siControl or si*BAF60c*. After 48 hours, cells were serum starved in Opti-MEM for 24 hours, and then ChIP-Seq was performed with Abs against BRG1, H3K9ac, and H3K27ac. IGV image showing BRG1, H3K9ac, and H3K27ac ChIP-Seq coverage at peaks within human *BCL2* gene promoter. (**G**) BRG1 and KLF5 binding to the predicted KLF-binding sites located in the *BCL2* promoter was determined by ChIP assay in HASMCs transfected with siControl or si*BAF60c* (30 nM) for 48 hours and serum starved in Opti-MEM for 24 hours. (**H**) A7r5 cells were transfected with WT or Del (region deleted) luciferase reporter driven by *BCL2* promoter and then infected with Ad-*lacZ* or Ad-*BAF60c*. After 48 hours, promoter activities were determined, and results are presented relative to A7r5 transfected with WT and infected with Ad-*lacZ* group (*n* = 8/group). (**I**) HASMCs were transfected with siControl or si*BAF60c* (30 nM). After 48 hours, cells were serum starved in Opti-MEM media for 24 hours, followed by nuclear protein isolation and Co-IP using BRG1 or KLF5 Abs. IgG was negative control. Three independent experiments were performed for **G** and **I**. Data are represented as mean ± SEM. Two-way ANOVA followed by Holm-Šidák post hoc analysis (**A**–**E** and **G**–**H**); paired *t* test (**I**).

Our in vivo data showed that *Baf60c* deficiency increased SMC apoptosis after Ang II infusion or elastase exposure. Accordingly, we found that *BAF60c* knockdown promoted HASMC death both in basal conditions and upon hydrogen peroxide (H_2_O_2_) or TNF-α plus cycloheximide (TNF-α+CHX) stimulation (3, 27), as measured by TUNEL staining (Figure 7A and Supplemental Figure 8A). To further investigate the potential mechanism underlying the antiapoptotic effect of BAF60c in VSMCs, we assessed the expression of B cell lymphoma 2 (BCL2), a crucial antiapoptotic protein. We found that knockdown of *BAF60c* reduced BCL2 expression in HASMCs in the presence or absence of H_2_O_2_ or TNF-α+CHX (Figure 7, B and C, and Supplemental Figure 8, B and C), while *BAF60c* overexpression significantly elevated BCL2 expression (Figure 7, D and E, and Supplemental Figure 8, D and E). In addition, our ChIP-Seq data showed a significantly reduced signal for BRG1, associated with diminished enrichment of H3K9ac, within the *BCL2* promoter (Figure 7F). Surprisingly, multiple Krüppel-like factor–binding (KLF-binding) sites were found within the BRG1 peak at the *BCL2* promoter. Reduced binding of BRG1 to those KLF-binding sites (–1099/–1135bp) was determined by ChIP assay in HASMCs upon *BAF60c* knockdown (Figure 7G). KLFs are a subclass of zinc finger DNA-binding transcription factors that are key regulators of cell growth and apoptosis (28). Among them, KLF5 has been identified as an essential regulator of arterial SMC proliferation and apoptosis upon injury (29). Accordingly, we found reduced KLF5 signal in those predicted KLF-binding sites within the *BCL2* promoter upon *BAF60c* knockdown (Figure 7G). In addition, we generated a *BCL2* promoter–driven luciferase reporter construct and found that BAF60c increased luciferase activity, while knockdown of KLF5 or deletion of the predicted KLF-binding site diminished the BAF60c-dependent effect (Figure 7H). Of note, BAF60c knockdown did not alter KLF5 expression (Supplemental Figure 8F) and KLF5 overexpression could not rescue the reduced BCL2 expression in HASMCs upon *BAF60c* knockdown (Supplemental Figure 8, G and H), but KLF5 knockdown significantly decreased the elevation of BCL2 in Ad-*BAF60c*–infected HASMCs (Supplemental Figure 8I). Thus, our data suggest that BAF60c-dependent transcriptional activation of BCL2 is associated with the interaction of the SWI/SNF complex and KLF5 instead of the transcriptional regulation of KLF5. Accordingly, KLF5 association with the SWI/SNF complex was reduced, while KLF5-HDAC2 interaction was enhanced upon *BAF60c* knockdown (Figure 7I). These results suggest that the SWI/SNF complex containing BAF60c forms a chromatin remodeler with KLF5, which, in turn, transcriptionally activates BCL2 expression.

To gain a more comprehensive understanding of the regulatory mechanisms of BAF60c in AAA, we measured the expression of contractile gene *Myh11*, inflammatory genes *Ccl2* and *Il6*, and the antiapoptotic gene *Bcl2* in the suprarenal abdominal aortas from *Baf60c^fl/fl^* *Apoe^–/–^* and *Baf60c*^SMKO^ *Apoe^–/–^* mice after saline or Ang II (1,000 ng/kg/min) infusion for 7 days. Consistent with the in vitro data, *Baf60c* KO significantly reduced *Myh11* and *Bcl2* expression, but increased *Ccl2* and *Il6* expression in the Ang II–infused aorta (Supplemental Figure 9). These data further demonstrate a protective role of BAF60c against VSMC dysfunction and apoptosis in response to a stimulus relevant to AAA.

## Discussion

VSMC homeostasis is controlled by a network of transcriptional regulators, including transcription factors (e.g., SRF), cofactors (e.g., myocardin, P300), and epigenetic regulators (e.g., SWI/SNF complex, HDACs) (7, 8, 19, 30, 31). These regulators form chromatin-remodeling complexes through physical interaction and are recruited to the regulatory regions of target genes to precisely regulate SMC-specific gene expression (7, 32, 33). The SWI/SNF complex containing a BRG1 ATPase subunit is required for SMC-specific gene expression (7, 8). However, the mechanism underlying the SWI/SNF complex’s selective regulation of VSMC homeostasis and vascular diseases, such as AAA, remains unclear. The BAF60 family, comprising BAF60a, BAF60b, and BAF60c, is unique in the SWI/SNF complex and serves, in a mutually exclusive fashion (i.e., if one BAF60 member is in the SWI/SNF complex, then neither of the other 2 is present), as a link between the SWI/SNF complex and transcription factors to regulate defined gene expression (9, 10, 34). However, the specific role of BAF60c association with the SWI/SNF complex in chromatin remodeling in VSMCs and pathologic changes in the aorta remained unexplored. Surprisingly, we found here that BAF60c is decreased in both human and murine AAA tissues and is the most abundant BAF60 subunit primarily expressed in VSMCs, as evidenced by reanalysis of our previously published scRNA-Seq data set (15). Our results further showed that loss of BAF60c in VSMCs promotes AAA formation in Ang II– and elastase-induced murine AAA models by inducing VSMC phenotypic switch and apoptosis and increasing vascular inflammation. We showed that BAF60c is essential for precise control of VSMC homeostasis, acting by serving as an SRF coactivator to preserve the VSMC contractile phenotype and as an NF-κB/P65 repressor to prevent vascular inflammation in response to pathologic stimuli. In addition, we found that BAF60c inhibits VSMC apoptosis by modulating antiapoptotic protein BCL2 expression. Overall, our data provide evidence that BAF60c-dependent chromatin remodeling concurrently regulates VSMC phenotypic switch and apoptosis, two hallmark features of AAA (3, 4, 35).

The SWI/SNF complex containing BRG1 is required for myocardin and myocardin-related transcription factor A (MRTFA) to regulate chromatin accessibility and induce SM-specific gene expression in SMCs and nonmuscle cells (7, 8). The BAF60 subunit serves as a bridge between the SWI/SNF complex and specific transcription factors (9, 10, 36). However, the 3 subunits’ role in regulating chromatin accessibility at the regulatory regions of VSMC-specific genes remains largely unexplored. Here, we showed that BAF60c is required for BRG1 to interact with SRF and induce VSMC-specific contractile gene expression, indicating BAF60c-dependent epigenetic regulation for preservation of the VSMC contractile phenotype. Consistently, BAF60c was demonstrated to interact with SRF and induce SMC genes in multipotent adult progenitor cells (14). Conversely, we found that BAF60c overexpression can increase the expression of SMC contractile markers, not only the early SMC markers, such as α-actin and SM22α. These findings deepen our understanding of the role of BAF60c in VSMC differentiation and modulation. The establishment of an epigenetic landscape is also essential for the binding of transcription factors to chromatin regulatory regions and for keeping an active or repressive chromatin state (9, 10, 37, 38). Epigenetic modifications, such as methylation and acetylation of histone H3 and H4, regulate the binding activity of SRF to the promoters of VSMC-specific genes (19). Our data indicate that BAF60c serves as a link between the SWI/SNF complex and SRF and that BAF60c-mediated chromatin remodeling potentiates chromatin accessibility and SRF-binding activity to VSMC-specific genes. These findings shed light on the comprehensive understanding of the epigenetic regulation of VSMC homeostasis and the contribution of BAF60c-mediated chromatin remodeling to VSMC biology. In contrast, BRG1 was demonstrated to be upregulated in the thoracic aortic aneurysm (TAA), and BRG1, in cooperation with long, noncoding RNAs MALAT1 and HIF1 α-antisense RNA1, was shown to mediate the epigenetic regulation of VSMC dysfunction and apoptosis in TAA (33, 39, 40). These data show how the SWI/SNF complex can work as both a positive and negative modulator of transcription in VSMCs during AAA and TAA development. BRM, another ATPase subunit of the SWI/SNF complex, can directly associate with myocardin to induce the expression of SM-specific genes in vitro and is required for MRTFA to induce SM-specific genes in nonmuscle cells (7, 8). Further follow-up studies, complementary to those here, will be required to clarify the role of the SWI/SNF complex containing BAF60c-BRM, its differences from the BAF60c-BRG1 complex in regulating VSMC biology, and the potentially specific contributions of both the BAF60c-BRG1– and BAF60c-BRM–containing SWI/SNF complexes to different cardiovascular diseases.

The NF-κB family is critical for the inducible expression of inflammatory genes in response to environmental cues (24). The chromatin status at NF-κB–binding sites is a major determinant in the binding of NF-κB and its cofactors and subsequent recruitment of chromatin-remodeling complexes, such as the SWI/SNF complex (24, 25, 41). P300/CBP histone acetyltransferase is one of the best-characterized coactivators for NF-κB, specifically the P65 subunit. The interaction of P65 and P300 results in the acetylation of the surrounding histones and subsequent transcriptional induction (36). Conversely, chromatin-repressive modifications or binding of corepressors is essential for maintaining a basal repressive state at the NF-κB target genes (25, 26). HDAC1, in addition to the removal of histone acetylation, functions explicitly as a corepressor of P65 dimers and P50 dimers to restrain the expression of inflammatory genes in the basal state (25). Although many of these cofactors are essential for the regulation of NF-κB target genes, the mechanisms of how NF-κB selectively recruits and interacts with them remain unclear. In our previous study, we defined BAF60a as a P65 coactivator, promoting the interaction of the SWI/SNF complex with P65 through P300 and, in turn, enhancing the expression of NF-κB target genes upon stimulation (36, 42). Conversely, our current study demonstrates that BAF60c, as a P65 repressor, functions to keep a high level of H3K9me2 and to promote the association of P65 and its repressor HDAC1 in VSMCs in the basal condition, thus showing an opposing effect to BAF60a on VSMC inflammation. These findings suggest that the SWI/SNF complex containing different BAF60 subunits is an essential determinant for NF-κB–selective interaction with different cofactors to precisely control the expression of its target genes. These findings are also consistent with the mutually exclusive nature of the assembly of different BAF60 subunits within the SWI/SNF complex (9) and define what we believe is a new contextual layer of regulation of the VSMC phenotype. Although these studies deepen our understanding of the chromatin dynamics and the regulatory mechanisms underlying NF-κB–mediated transcriptional programs, numerous transcription factors and chromatin-modifying complexes govern the specificity of NF-κB–mediated transcription. Their roles and relationship with NF-κB and the potential dependence of their regulatory effects on the BAF60 family remain to be further explored. Additionally, SRF, myocardin, and MRTFA suppress inflammatory gene expression in cultured SMCs (43, 44) and BRG1 is required for myocardin and MRTFA-induced SMC gene expression (7, 8), suggesting that BAF60c-SRF interaction may also have an antiinflammatory impact on VSMCs.

VSMC phenotypic switch has already been identified in vivo during AAA through histological and lineage-tracing studies (18, 35). Accordingly, in our study, we confirmed the existence of SMC-derived macrophage-like cells in the aneurysmal aorta by immunofluorescence costaining, although we acknowledge that it is not as rigorous an evaluation to verify VSMC phenotypic switch as combining SMC-specific Baf60c KO in lineage-tracing mouse models would be. In further support of our costaining data, our findings show that BAF60c protects against AAA formation through preservation of VSMC contractile phenotype and inhibition of VSMC inflammatory activation. As such, loss of BAF60c should result in VSMC phenotypic switch and transition to other phenotypes, such as macrophage-like or mesenchymal-like cells, contributing to AAA development. Additionally, the mechanism by which BAF60c modulates VSMC phenotype switching to different cell types in the aorta will be addressed in follow-up studies.

VSMC apoptosis is limited in the healthy aorta, but becomes prominent during AAA development. Inhibition of VSMC apoptosis remains a promising strategy for AAA treatment (3). Here, we showed that, both in vivo and in vitro, loss of BAF60c in VSMCs markedly increased VSMC apoptosis, thus revealing another critical protective effect of BAF60c on VSMC survival. Specifically, we demonstrated that BAF60c is required to recruit the SWI/SNF complex to the BCL2 promoter and subsequently engage in a regulatory interaction with KLF5 to activate BCL2 expression in VSMCs, thus uncovering a BAF60c-dependent antiapoptotic regulation to prevent VSMC loss during AAA development. Seventeen members of the KLF family have been identified in the mammalian genome, and most of them have been shown to regulate cell proliferation, differentiation, and apoptosis (28, 29). Here, we identified KLF-binding sites within the BCL2 promoter and demonstrated the BAF60c-dependent interaction of KLF5 and BRG1. It is possible that this regulatory interaction may be recapitulated in aneurysms in general, since lower BCL2 expression has been associated with VSMCs in a variety of aneurysms, including abdominal and thoracic aortic and intracranial (3, 45, 46). Moreover, SRF was demonstrated to promote BCL2 expression and prevent SMC apoptosis through interaction with chromodomain helicase DNA-binding protein 8 (CHD8) (47, 48). It will be relevant to investigate in future studies whether BAF60c or the SWI/SNF complex contributes to the regulatory effect of SRF on BCL2 expression and SMC survival.

Despite being ubiquitously expressed, BRG1 has been shown to selectively regulate gene expression through its interaction with specific transcription factors (7, 8). Our findings show that BAF60a and BAF60c are selectively and differentially required for transcriptional activation of the genes related to VSMC inflammation and contractile phenotype in the context of AAA. These results suggest that BAF60 subunits serve, in a mutually exclusive fashion, as a bridge between BRG1 and specific transcription factors, which can explain, at least in part, the mechanism underlying BRG1-selective regulation of gene expression in VSMCs. Additionally, we found downregulation of BAF60c, in contrast with upregulation of BAF60a, in both human and murine AAA samples (36). This underscores the relevance of changes in the global epigenome mediated by the BAF60 family in AAA and supports the concept that dynamic changes in epigenetic controls can alter AAA manifestation and outcomes. Those findings show that the distinct expression patterns and regulatory mechanisms of different BAF60 subunits likely operate in a dynamic equilibrium in VSMCs that is disrupted in vascular diseases to alter the control of the VSMC transcriptome and, consequently, VSMC homeostasis.

In summary, we demonstrate that BAF60c is a protective factor against VSMC dysfunction and that its loss aggravates AAA. Mechanistically, BAF60c preserves VSMC homeostasis by serving as an SRF coactivator to maintain the VSMC contractile phenotype and acts as a P65 repressor to prevent vascular inflammation in response to pathologic stimuli. Furthermore, we showed that BAF60c is required for KLF5-dependent regulation of BCL2 expression in VSMCs. These findings shed light on the comprehensive understanding of how BAF60c contributes to maintaining VSMC homeostasis and indicate that promoting or preserving VSMC resistance to pathologic cues by modulating epigenetic modifications mediated by the BAF60 family may eventually lead to pharmacological interventions for AAA and other cardiovascular diseases.

## Methods

### Materials and reagents.

Rabbit Abs for BAF60c (catalog ab171075), SM α-actin (catalog ab119952), calponin (catalog ab46794), SM22α (catalog ab103135), BRG1 (catalog ab110641), and BCL2 (catalog ab182858) were purchased from Abcam. CD45 Abs (catalog 550539) were purchased from BD Biosciences. Mac2 Abs (catalog 14-5301-85), rhodamine phalloidin (catalog R415), and myocardin Abs (catalog PA5-67810) were purchased from Thermo Fisher Scientific. Abs against SRF (catalog 5147), P300 (catalog 70088), BAF155 (catalog 11956), BAF170 (catalog 12760), BAF47 (catalog 91735), P300(catalog 54062), NF-κB P65 (catalog 8242), phosphorylated NF-κB P65 (p-P65, catalog 3033), phosphorylated IKKα/β (p-IKKα/β) (catalog 2697), IKKα (catalog 11930), NF-κB1 P105/P50 (catalog 13586), acetyl-histone H3 (Lys9) (H3K9ac, catalog 9649), di-methyl-histone H3 (Lys9) (H3K9me2, catalog 4658), acetyl-histone H3 (Lys27) (H3K27ac, catalog 8173), tri-methyl-histone H3 (Lys27) (H3K27me3, catalog 9733), HDCA1 (catalog 5356), HDAC2 (catalog 5113), KLF5 (catalog 51586), β-actin (rabbit mAb, catalog 4970; and mouse mAb, catalog 3700), GAPDH (catalog 5174), and rabbit IgG (catalog 2729) were purchased from Cell Signaling Technology (CST). Ang II (catalog H-1706) was from Bachem. Recombinant human TGF-β1 (catalog 100-21C) was from PeproTech. Recombinant human PDGF-BB (catalog 220-BB-010) and TNF-α (catalog 210-TA) were from R&D Systems. Cycloheximide (14126) was from Cayman Chemical. Hydrogen peroxide (30% in water, BP2633500) was from Thermo Fisher Scientific. Plasmid pcDNA3-*KLF5* (40917) was from Addgene.

### Human aortic samples.

Detailed information for human aortic tissues from AAA patients or organ donors is given in Supplemental Table 1.

### Animals and murine AAA models.

*Baf60c^fl/fl^* mice on a C57BL/6J background were described previously (49). In brief, exon 2 of the mouse *Baf60c* gene was flanked with 2 *loxP* sites. *Myh11*-CreER^T2^ (50) (strain 019079) and *Apoe^–/–^* mice (B6.129P2-Apoe^tm1Unc/J^, strain 002052) were purchased from Jackson Laboratory. *Myh11*-CreER^T2^ *Baf60c^fl/fl^* mice on a C57BL/6J background were generated by crossbreeding female *Baf60c^fl/fl^* mice with male *Myh11*-CreER^T2^ mice. *Myh11*-CreER^T2^ *Baf60c^fl/fl^* mice were next crossbred with *Apoe^–/–^* mice to generate *Myh11*-CreER^T2^ *Baf60c^fl/fl^* *Apoe^–/–^* mice. Because the Cre transgene was inserted into the Y chromosome (50), only male *Myh11*-CreER^T2^ *Baf60c^fl/fl^* mice could be generated, and accordingly, only male mice were used in this study. Male *Myh11*-CreER^T2^ *Baf60c^fl/fl^* and *Baf60c^fl/fl^* mice with or without *Apoe^–/–^* were intraperitoneally injected with tamoxifen (MilliporeSigma, T5648, 75 mg/kg/d) for 5 consecutive days to generate VSMC-specific *Baf60c*-KO (*Baf60c*^SMKO^, *Baf60c*^SMKO^ *Apoe^–/–^*) and floxed control (*Baf60c^fl/fl^*, *Baf60c^fl/fl^* *Apoe^–/–^*) mice for the current study. Mice were allowed 9 days to clear the excess tamoxifen and subjected to murine AAA models.

The *Pcsk9*/Ang II–induced AAA model was implemented as previously described (3, 36, 51). Briefly, 8- to 10-week-old male C57BL/6J mice were injected intraperitoneally with 2 × 10^11^ genomic copies of adeno-associated virus carrying a gain-of-function mutation of mouse *Pcsk9* (AAV-*Pcsk9.D377Y*, Penn Vector Core, University of Pennsylvania, Philadelphia, Pennsylvania, USA) and switched to a Western diet containing 0.2% cholesterol by weight (TD.88137, Envigo) to induce hypercholesterolemia. Two weeks after AAV injection, Ang II (Bachem, H-1706, 1000 ng/kg/min) was subcutaneously infused into mice via mini-pumps (Alzet, model 2004) for 4 weeks. The suprarenal abdominal aortas for Western blot were perfused with PBS+2% heparin to remove excessive blood, followed by protein extraction.

The Ang II–induced AAA model was implemented as previously described (17, 52). Briefly, 16-week-old male *Baf60c*^SMKO^ *Apoe^–/–^* and *Baf60c^fl/fl^* *Apoe^–/–^* mice were subcutaneously implanted with mini-pumps (Alzet, model 2004) to infuse Ang II (1000 ng/kg/min) for 4 weeks. Blood pressure was monitored weekly by a noninvasive tail-cuff method (Visitech BP-2000), as previously described (53).

The elastase-induced AAA model was implemented as previously described (54, 55). In brief, 10- to 12-week-old male *Baf60c*^SMKO^ and *Baf60c^fl/fl^* mice were anesthetized by intraperitoneal injection of a mixture of ketamine (100 mg/kg) and xylazine (5 mg/kg). The infrarenal abdominal aorta was isolated and then surrounded with a sterile gauze presoaked with 30 μL of elastase (44 units/mL, MilliporeSigma, E1250). After 30 minutes of incubation, the gauze was removed, and the abdominal cavity was washed twice with sterile saline before suturing. Blood pressure was monitored weekly by tail cuff using Visitech BP-2000.

Two weeks after elastase exposure or 4 weeks after Ang II infusion, mice were euthanized and blood was collected by ventricle puncture before perfusion with saline and 10% buffered formalin in PBS through the left ventricle to remove the remaining blood; this was followed by isolation of the aortas for ex vivo measurements. The maximum external diameters of infrarenal abdominal aortas from the elastase-induced AAA model and suprarenal abdominal aortas from the Ang II–induced AAA model were determined. Maximum diameters 50% larger than those of the adjacent portion were considered as AAA (4, 36). The mice with rupture of the thoracic aorta were excluded from the calculation of Ang II–induced AAA incidence.

Plasma total cholesterol (TC) and triglyceride (TG) levels were measured at the end point by enzymatic kits (Wako Diagnostics). Serum MCP-1 and IL-6 levels were determined by ELISA at the Immunology Core at the University of Michigan.

### Adenovirus infection of mouse suprarenal abdominal aorta.

The mouse suprarenal abdominal aorta was infected with adenovirus through periadventitial delivery. Briefly, the mouse upper abdomen was open, and the suprarenal abdominal aorta was isolated. At this point, 50 μl of 22.5% w/v pluronic F-127 gel (56) loaded with 2 × 10^9^ viral particles of Ad-*GFP* or Ad-*BAF60c* were placed around the suprarenal abdominal aorta. Before and 2 weeks after adenovirus infection, the aortic diameter was measured by ultrasound imaging at the Physiology and Phenotyping Core of the University of Michigan Medical Center.

### Cell culture.

HASMCs (CC-2571) from a 22-year-old male were purchased from Lonza and cultured in SMC growth medium 2 (Lonza, CC-3182) containing 5% fetal bovine serum (Lonza) and 1% penicillin/streptomycin solution (Gibco, Thermo Fisher Scientific; 15140122) at 37°C, 5% CO_2_ in a humidified cell culture incubator. HASMCs were used from passages 4 to 8 in all experiments. The rat embryonic thoracic aortic SMC line A7r5 was purchased from ATCC and cultured in DMEM/Nutrient Mixture F-12 (DMEM/F12) (Gibco, Thermo Fisher Scientific; 11320-033) containing 10% FBS (Thermo Fisher Scientific) and 50 mg/mL of a penicillin/streptomycin solution. Mouse BMDMs were obtained as previously described (57).

### Histology.

The suprarenal abdominal aortas from the Ang II–induced AAA model and the infrarenal abdominal aortas from the elastase-induced AAA model were excised. Human and murine aortic samples were fixed with 10% buffered formalin in PBS and then embedded in paraffin at the In Vivo Animal Core (IVAC) at the University of Michigan. The serial sections (5 μm thick, 200 μm apart) were deparaffinized, rehydrated, and stained with a Verhoeff–van Gieson (VVG) staining kit (Electron Microscopy Sciences) according to the manufacturer’s instructions for elastin assessment. Based on previous reports (4, 55), elastin degradation was graded as follows: 1, <25% degradation; 2, 25% to 50% degradation; 3, 50% to 75% degradation; and 4, >75% degradation.

For immunostaining, the rehydrated sections were boiled for 15 minutes in citrate buffer (pH 6.0) (Invitrogen, 00-5000) for epitope retrieval. After blocking with 5% donkey serum in PBS for 1 hour at room temperature, the sections were incubated with primary Abs against BAF60c (Abcam, catalog ab171075, 1:100 dilution), SM22α (Abcam, catalog ab103135, 1:100 dilution), CD45 (BD Biosciences, catalog 550539,1:50 dilution), or Mac2 (Thermo-Fisher Scientific, catalog 14-5301-85, 1:100 dilution) at 4°C overnight. The negative controls were sections incubated with species-matched IgG. After washing with PBS, the sections were incubated with Alexa Fluor–conjugated secondary Abs (Jackson ImmunoResearch Laboratories) for 1 hour at room temperature. After washing with PBS, slides were mounted with ProLong Gold Antifade Mountant with DAPI (Invitrogen, P36935) before image collection with an Olympus DP73 microscope. The numbers of infiltrated leukocytes (CD45^+^) and macrophages (Mac2^+^) in the aortic wall and the percentages of SM22α^+^Mac2^+^ cells in the media were measured with ImageJ software (NIH), and an average number of 3 to 5 sections (4 random fields per section) for each mouse was calculated.

### Apoptosis assay.

The DeadEnd Fluorometric TUNEL system (Promega, G3250) was used to detect cell apoptosis according to the manufacturer’s protocol. Briefly, to detect cell apoptosis in paraffin-embedded aortic tissue, the rehydrated tissue sections (5 μm thick) were incubated in 0.85% NaCl for 5 minutes at room temperature before fixation in 4% methanol-free formaldehyde solution for 15 minutes at room temperature. Next, the sections were permeabilized in proteinase K solution (20 μg/mL) for 10 minutes at room temperature and fixed in 4% methanol-free formaldehyde solution for 5 minutes at room temperature, followed by incubation with equilibration buffer for 10 minutes at room temperature. Terminal deoxynucleotidyl transferase (TdT) reaction mixture was added to the aortic sections for 60 minutes with incubation at 37°C, followed by 2× SSC buffer to stop the reaction. Slides were mounted with ProLong Gold Antifade Mountant with DAPI (Invitrogen, P36935), and green fluorescence of apoptotic cells within the aortic wall was captured with an Olympus DP73 microscope. Quantification of the percentage of TUNEL^+^ cells in the media was performed with ImageJ software, and an average number of 3 to 5 sections (4 random fields/section) for each mouse were calculated.

For analysis of apoptotic HASMCs, cells were fixed in 4% methanol-free formaldehyde solution for 25 minutes at 4°C and permeabilized in 0.2% Triton X-100 solution in PBS for 5 minutes. Next, cells were incubated in equilibration buffer, TdT reaction buffer, and 2× SSC buffer and subsequently mounted with ProLong Gold Antifade Mountant with DAPI as described above.

### Isolation and culture of MASMCs.

Mouse primary aortic SMCs (MSMCs) were isolated from 8-week-old male *Baf60c^fl/fl^* and *Baf60c*^SMKO^ mice as previously described (58). Briefly, aortas were harvested after removing the perivascular adipose tissue and digested in 1 mg/mL type 2 collagenase (Worthington Biochemical Corporation, LS004174, ≥125 units/mg) in HBSS (Invitrogen, 14170-112) at 37°C for 10 minutes. After removing the adventitial layer, the remaining aortic tissue was minced and digested in enzymatic solution (1 mg/mL type 2 collagenase and 0.5 mg/mL elastase [MilliporeSigma, E-0258, ≥4.0 units/mg] in HBSS) for 1 hour at 37°C. After digestion, MSMCs were dissociated, washed, and cultured in DMEM/F12 supplemented with 20% FBS and 1% penicillin/streptomycin. Passages 4 to 7 of MSMCs were used for experiments.

### Plasmid constructs.

A DNA fragment of human *BCL2* promoter (–1670/+16) was PCR amplified and cloned into the pGL4.11 vector (Promega). Deletion of the predicted KLF-binding site (–1125/–1099) within the cloned *BCL2* promoter fragment (–1670/+16) was performed using the In-Fusion HD Cloning Plus Kit (TaKara Bio, 638910). All PCR products were validated by DNA-Seq.

### Luciferase assay.

A7r5 cells at 80% confluence were transfected with the indicated luciferase plasmids using Lipofectamine 2000 (Thermo Fisher Scientific, 11668019) according to the manufacturer’s protocol. After 12 hours, the cells were infected with Ad-*lacZ* and Ad-*BAF60c* (10 MOI). Forty-eight hours after infection, luciferase reporter activity was measured by dual-luciferase assay (Promega, E1910) and normalized against Renilla activity.

### siRNA and plasmid transfection.

HASMCs were transfected with 30 nM si*BAF60c* (Thermo Fisher Scientific, s13158), si*KLF5* (Thermo Fisher Scientific, s2115), and si*HDAC1* (Thermo Fisher Scientific, s73), and A7r5 cells were transfected with 30 nM si*Baf60c* (Thermo Fisher Scientific, 252172), si*Klf5* (Thermo Fisher Scientific, s136575), or Silencer Select Negative Control siRNA (siControl, Thermo Fisher Scientific, 4390843). For both HASMCs and A7r5 cells, Lipofectamine RNAiMAX Reagent (Invitrogen, 13778150) was used for transfection according to the manufacturer’s instructions.

HASMCs were transfected with 30 nM si*BAF60c* or siControl and pcDNA3.1 or pcDNA3-*KLF5*-*FLAG* (6-well plate) using Lipofectamine 2000 Transfection Reagent (Thermo Fisher Scientific, 11668019) according to the manufacturer’s instructions.

### Total RNA isolation and quantitative real-time qPCR analysis.

Total RNA from HASMCs or A7r5 cells was extracted using the RNeasy Mini Kit (QIAGEN, 74106) according to the manufacturer’s instructions. The SuperScript III First-Strand Synthesis System (Thermo Fisher Scientific, 18080051) and random primers were used to reverse transcribe RNA into cDNA. Gene expression was quantified by the Real-Time PCR Detection System (Bio-Rad) using iQ SYBR Green Supermix (1708882, Bio-Rad). The gene expression level was normalized to the internal control β-actin. The primer sequences used are listed in Supplemental Table 4.

### RNA-Seq.

Total RNA was extracted from HASMCs after transfection with siControl or si*BAF60c* (*n* = 3/group) using the RNeasy Mini Kit (QIAGEN, 74106) and then treated with RNase-free DNase I (QIAGEN, 79254) according to the manufacturer’s instructions. RNA library preparation and sequencing were performed by the Advanced Genomics Core at the University of Michigan. In brief, RNA quality was determined by BioAnalyzer (Agilent). Then the RNA library was prepared with the NEBNext Ultra RNA Library Prep Kit (New England Biolabs), and 51 bp read paired-end sequencing was performed on a HiSeq 6000 platform (Illumina). A total of 266 million reads were generated, with an average of 38 million reads per sample. RNA-Seq read mapping was performed as described previously (59). In brief, FastQC (Babraham Bioinformatics) was used for quality control of the sequencing reads from each sample. Gene expression quantification was performed using Salmon, version 0.14.0 (60), with human cDNA sequences of GRCh38 (Ensembl database) as reference. Differential expression analysis was performed with the DeSeq2 package in R (61). GSEA (23, 62) was performed to interpret gene expression profiles of siControl and si*BAF60c* transfected HASMCs. Genes were mapped to the HALLMARK and GO gene set in the MSigDB for pathway analysis. The RNA-Seq raw data and processed≈data described in the article have been deposited in the NIH’s Gene Expression Omnibus database (GEO GSE180586).

### ELISA.

HASMCs were transfected with siControl or si*BAF60c* or infected with Ad-*lacZ*, Ad-*BAF60c*. After 48 hours, cells were cultured in opti-MEM with or without 20 ng/mL TNF-α for 24 hours. The cell culture media were collected for quantification of MCP-1 and IL-6 by ELISA in the Immunology Core at the University of Michigan.

### Macrophage migration assay.

HASMCs were seeded in the lower chambers of a Corning Costar Transwell (6.5 mm diameter, 8.0 μm pore size, MilliporeSigma, CLS3464) plate and transfected with siControl or si*BAF60c* or infected with Ad-GFP or Ad-*BAF60c*. After 48 hours, the cells were incubated in fresh opti-MEM (Gibco, Thermo Fisher Scientific; 31985-070) with or without TNF-α (20 ng/mL) stimulation for 4 hours. The medium was subsequently changed to fresh opti-MEM. BMDMs were seeded in the upper chambers of the Transwells (10^5^/chamber) and cocultured with HASMCs in the fresh opti-MEM. After 12 hours of coculture, the BMDMs on the upper surface of the Transwell insert were removed by scraping with a cotton swab, and the membranes were fixed in methanol for 30 minutes before staining with 0.1% crystal violet (MilliporeSigma, C0775) for 20 minutes at room temperature. Images of 4 random fields per Transwell were captured by microscopy, and the numbers of migrated cells were calculated using ImageJ software.

### Nuclear protein extraction.

Nuclear extracts for HASMCs and A7r5 cells were prepared as previously described (63). Briefly, cells were lysed on ice for 20 minutes with EB0 hypotonic buffer (50 mM Tris, pH 7.5, 0.1% NP-40, 1 mM EDTA, 1 mM MgCl_2_ supplemented with the cOmplete EDTA-free protease inhibitor cocktail [Roche, 11873580001] and PhosSTOP phosphatase inhibitor [Roche, 4906845001]). Lysates were pelleted at 2,400*g* for 5 minutes at 4°C. Supernatants were discarded, and nuclei were resuspended in EB300 high-salt buffer (50mM Tris, pH 7.5, 300 mM NaCl, 1% NP-40, 1 mM EDTA, 1 mM MgCl_2_ supplemented with the protease inhibitor cocktail and phosphatase inhibitor). Lysates were incubated on ice for 20 minutes, followed by centrifugation at 6,000*g* for 10 minutes at 4°C. Supernatants were collected, and protein concentrations were quantified. Finally, nuclear extracts were supplemented with 1 mM DTT.

### Co-IP.

200 μg of nuclear extracts from HASMCs or A7r5 cells were incubated with 2 μg of primary Abs against BRG1 (Abcam, catalog ab110641), SRF (CST, catalog 5147), P300 (CST, catalog 70088), P65 (CST, catalog 8242), KLF5 (CST, catalog 51586), or normal rabbit IgG (CST, catalog 2729), rotating overnight at 4°C. The immunocomplexes were then precipitated with Protein A Magnetic Beads (CST, 73778) and eluted with loading buffer for Western blot analysis.

### Protein extraction and Western blot.

Cells were lysed in RIPA lysis buffer (Thermo Fisher Scientific, 89901) supplemented with cOmplete EDTA-free protease inhibitor cocktail (Roche, 11873580001) and PhosSTOP phosphatase inhibitor (Roche, 4906845001). Human and mouse tissues were homogenized in T-PER tissue protein extraction reagent (Thermo Fisher Scientific, 78510). Cells or tissues were lysed at 4°C for 30 minutes and centrifuged at 6,000*g* for 15 minutes to remove insoluble debris. Protein extracts were resolved in 10% SDS-PAGE gels and transferred to nitrocellulose membranes (Bio-Rad, 1620115). Membranes were blocked in TBST containing 5% fat-free milk for 1 hour at room temperature and incubated with primary Abs at 4°C overnight. After washing 3 times with 1× TBST, membranes were incubated with secondary Abs (1:10,000 dilution, Li-Cor Bioscience) for 1 hour at room temperature. After 3 washes with 1× TBST, bands were scanned using the Odyssey Imaging System (Li-Cor Bioscience) and quantified with the LI-COR Image Studio Software.

### ChIP assay.

ChIP assays were performed using the SimpleChIP Enzymatic Chromatin IP kit (Magnetic Beads) (CST, 9003S) according to the manufacturer’s protocol. In brief, HASMCs were transfected with siControl or si*BAF60c*. A7r5 cells were transfected with siControl or si*Baf60c*. After 48 hours, cells were cultured in opti-MEM media for an additional 24 hours. Next, cells were treated with 2 mM disuccinimidyl glutarate (DSG) (Thermo Fisher Scientific, 20593) at room temperature for 45 minutes to crosslink the SWI/SNF complex with DNA and then incubated with 1% fresh paraformaldehyde at room temperature for 10 minutes to crosslink the histone/transcription factor complexes with DNA, followed by 0.1% glycine incubation at room temperature for 5 minutes. The nuclei pellets were digested with Micrococcal Nuclease at 37°C for 20 minutes, followed by sonication (Branson Sonifier SLPe, 20 seconds of 35% amplification, 3 times). After centrifugation at 9,400*g* for 10 minutes at 4°C, chromatin was immunoprecipitated with Abs against SRF (1:50 dilution, CST, catalog 5147), BRG1 (1:50 dilution, Abcam, catalog ab110641), H3K9ac (1:50 dilution, CST, catalog 9649), H3K9me2 (1:50 dilution, CST, catalog 4659), or normal rabbit IgG (CST, catalog 2729) at 4°C overnight with gentle rotation. The protein/DNA complexes were immunoprecipitated by ChIP grade protein G magnetic beads with rotation for 2 hours at 4°C, followed by 3 washes in low-salt buffer and 1 wash in high-salt buffer and elution at 65°C for 30 minutes. The eluted protein-DNA complexes were reversed with proteinase K at 65°C for 2 hours. DNA was purified and then amplified by real-time qPCR with the following primers targeted to the rat *Myh11* promoter (–432/–355): forward primer: 5′-GACTTGATCAGCCTTCCTTC-3′ and reverse primer: 5′-AGGGCCTAGCCCATTGTACA-3′; rat *Acta2* promoter (–492/–349), forward primer: 5′-CACATTCTCATATGCTGCCCA-3′ and reverse primer: 5′-AGGGACATAGTGATTAGCACA-3′; rat *Cnn1* promoter (–306/–242), forward primer: 5′-TCCCCTACTGTGGACACTGA-3′ and reverse primer: 5′-GATCGTCCGGATCTCCTA-3′; rat *Tagln* promoter (–722/–598), forward primer: 5′-AGTTGAAGGCCAGCCTAGT-3′ and reverse primer: 5′-ATAGAGAGGGAAGTTGGACC-3′; human *MYH11* promoter (–692/–624), forward primer: 5′-GCAGCGCACAGTTAGACTTGA-3′ and reverse primer: 5′-GAAGAAGAAGCCGGAAGTAAGTG-3′; human *CCL2* promoter (–1833/–1684), forward primer: 5′-TCCACCAGAGTCTGAAATGGC-3′ and reverse primer: 5′- AGTAGATGTGCTGAGACTCCCA-3′; and human *BCL2* promoter (–1178/–1023), forward primer: 5′-AGAACTTCGTAGCAGTCATCCT-3′ and reverse primer: 5′- TGGATAAATGAAGGCAGGACGC-3′. ChIP-qPCR data were reported as percentage of input, which can be calculated by the following formula: (% input = 2^((Ct(input) – log2(dilution factor)) – Ct(IP))^ × 100 (64). The input sample used was 4% of the DNA amount, and thus the dilution factor is 25.

### ChIP-Seq.

Samples containing 30 ng of DNA were immunoprecipitated with Abs against BRG1 (1:50 dilution; Abcam, catalog ab110641), H3K9ac (1:50 dilution; CST, catalog 9649s), H3K27ac (1:50 dilution; CST, catalog 8173s), or H3K9me2 (1:50 dilution; CST, catalog 4659s), and their respective inputs were sent to the Advanced Genomics Core at the University of Michigan for quality control, library preparation, and sequencing. In brief, the DNA library was prepared using the NEBNext Ultra II FS DNA Library Prep Kit for Illumina (New England Biolabs) and sequenced on an Illumina NovaSeq 5000 S1 flow cell with 100 bp paired-end reads. An average of 40 million reads was generated for each sample. ChIP-Seq reads were aligned to the human genome (NCBI GRCh19) using bowtie2, version 2.3.5, with default settings. Peaks were called by MACS2 software, and the reads from input served as a control with the following parameters: -f BAMPE -g hs -B --SPMR -q 0.05. Samples were normalized to adjust for sequencing depth. Peaks were visualized in the Integrative Genomics Viewer (IGV). The coverage of ±3 Kb around each gene TSS was quantified in reads per million mapped reads (RPM) using Deeptools bamCoverage (65). ChIP-Seq signals detected for H3K9ac were calculated as relative read ratio per bin (a 10 bp sliding window) within TSS ± 3 Kb and plotted as box plots. The ChIP-Seq raw data and processed data described in the paper have been deposited in the GEO database (GSE180586).

### Immunofluorescence staining of cultured VSMCs.

HASMCs cultured in Falcon Chambered Cell Culture Slides (Fisher, 08774208) were fixed in 4% paraformaldehyde in PBS for 15 minutes at room temperature. After washing with PBS 3 times, slides were permeabilized in a permeabilizing solution (0.1% Triton X-100 and 0.5% BSA in PBS) for 10 minutes at room temperature and incubated in blocking buffer (5% donkey serum in PBS) for 1 hour at room temperature. Next, slides were stained with primary Abs against P65 (1:100, CST, catalog 8242) in dilution buffer (5% normal donkey serum in PBS) overnight at 4°C. After washing with PBS 3 times, slides were incubated with Alexa Fluor–conjugated secondary Abs (Jackson ImmunoResearch Laboratories) and subsequently mounted with ProLong Gold Antifade Mountant with DAPI (Invitrogen, P36935) before images were captured with an Olympus IX73 microscope.

### Statistics.

Statistical analyses were performed using GraphPad Prism 8.0 software (GraphPad Software) or RStudio (for RNA-Seq). Unless indicated otherwise, data are presented as mean ± SEM. All data were evaluated for normality and equal variance. For normally distributed data, Student’s *t* test was used to compare the differences between 2 groups, and 1-way ANOVA followed by Tukey’s post hoc analysis or 2-way ANOVA followed by Holm-Šidák post hoc analysis was used for comparison among 3 or more groups. For data that were not normally distributed, nonparametric tests, including Mann-Whitney *U* test, χ^2^ test, and Mantel-Cox test (survival percentage), were used to compare 2 groups. *P* < 0.05 was considered statistically significant. All results are representative of at least 3 independent experiments.

### Study approval.

All animal procedures were performed according to protocols approved by the Institutional Animal Care and Use Committee (IACUC) at the University of Michigan. The human aortic tissues used in this study were obtained from the Cardiovascular Health Improvement Project (CHIP) core of the Frankel Cardiovascular Center (CVC) at the University of Michigan with Institutional Review Board approval (Hum00052866) from the Human Research Protection Program and Institutional Review Boards of the University of Michigan Medical School. All AAA patients provided written, informed consent.

## Author contributions

GZ, YZ, ZC, and JZ performed the experiments and results analysis. GZ and JZ wrote the article. HL, HL, HW, WL, YL, TZ, OR, YG, LC, BY, MTGB, and JDL provided technical support and contributed to the discussion of the project. MTGB did the critical editing of the article. YEC and JZ designed the research and discussed the results.

## Supplementary Material

Supplemental data

Supplemental tables 1-4

## Version 1 09/06/2022

In-Press Preview

## Version 2 11/01/2022

Electronic publication
