# Supplementary material for: BAF60c prevents abdominal aortic aneurysm formation through epigenetic control of vascular smooth muscle cell homeostasis
Source: J Clin Invest. 2022 Nov 1;132(21):e158309. doi: 10.1172/JCI158309 (PMC9621131; doi:10.1172/JCI158309)
Supplement: Supplemental data [file jci-132-158309-s157.pdf]

Supplemental Data

BAF60c Prevents Abdominal Aortic Aneurysm Formation through Epigenetic Control of Vascular Smooth Muscle Cell Homeostasis

Guizhen Zhao[1], Yang Zhao[1], Haocheng Lu[1], Ziyi Chang[1], Hongyu Liu[1], Huilun Wang[1], Wenying Liang[1], Yuhao Liu[1], Tianqing Zhu[1], Oren Rom[1,2], Yanhong Guo[1], Lin Chang[1], Bo Yang[3], Minerva T. Garcia-Barrio[1], Jiandie D. Lin[4], Y. Eugene Chen[1], Jifeng Zhang[1]

Affiliations: [1]Frankel Cardiovascular Center, Department of Internal Medicine, University of Michigan Medical Center, Ann Arbor, MI 48109, USA; [2]Department of Pathology and Translational Pathobiology, Louisiana State University Health Science Center-Shreveport, Shreveport, LA 71103, USA; [3]Department of Cardiac Surgery, University of Michigan Medical Center, Ann Arbor, MI 48109, USA; [4]Life Sciences Institute and Department of Cell & Developmental Biology, University of Michigan, Ann Arbor, MI 48109, USA.

Corresponding Authors:

Jifeng Zhang, PhD, Frankel Cardiovascular Center, Department of Internal Medicine, University of Michigan Medical Center, NCRC Bldg26, Room 357S. 2800 Plymouth Rd, Ann Arbor, MI 48109. Email: jifengz@umich.edu

Y. Eugene Chen, MD, PhD, Frankel Cardiovascular Center, Department of Internal Medicine, University of Michigan Medical Center, NCRC Bldg26, Room 361S. 2800 Plymouth Rd, Ann Arbor, MI 48109. Email: echenum@umich.edu

The authors have declared that no conflict of interest exists.

Supplementary Figures

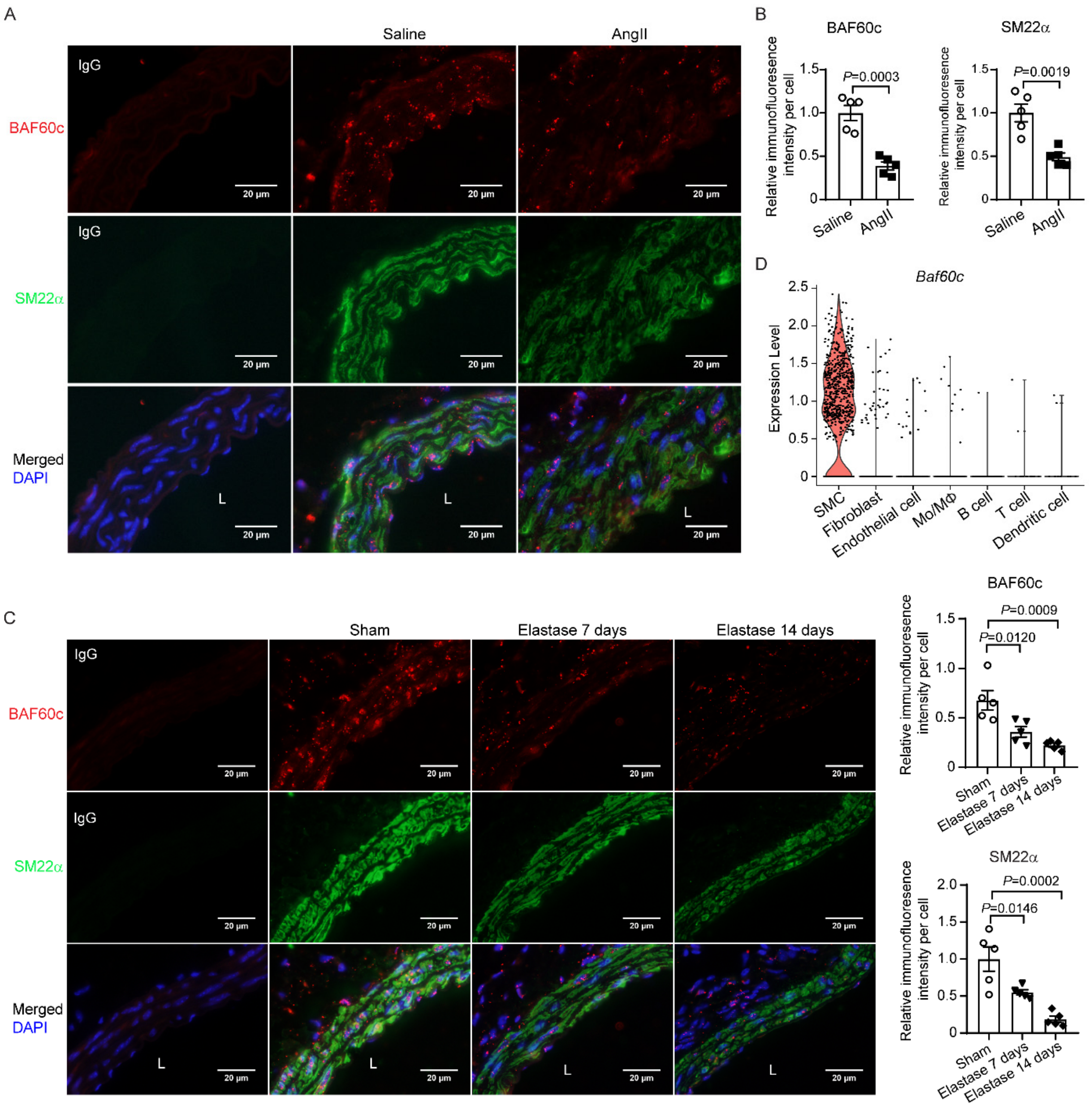

Supplementary Fig. 1. BAF60c is reduced in AAA tissues and selectively expressed in VSMC. **A-B**, Representative immunofluorescence staining (A) and quantification (B) of BAF60c (red) and SM22α (green) in the suprarenal abdominal aortas of C57BL/6J mice injected intraperitoneally with AAV-*Pcsk9.D377Y* and infused with saline or AngII (1,000 ng/kg/min) for 28 days. n=5/group. Nuclei stained with DAPI are blue. Scale bar=20μm.**C**, C57BL/6J mice

subjected to elastase-induced infrarenal AAA model. Sham, 14 days after heat-inactivated elastase exposure for 30min; Elastase, 7 days and 14 days after elastase exposure for 30 min (n=4/group). Representative immunofluorescence staining and quantification of BAF60c (red) and SM22α (green) in the infrarenal abdominal aortas (n=5/group). Nuclei stained with DAPI are blue. Scale bar=20μm. **D**, scRNA-seq analysis of the infrarenal abdominal aortas (pooled from 5 mice for each group) isolated from 10-week-old C57BL/6J mice. The Violin plot shows *Baf60c* expression by cell populations. Student's *t*-test for B-C.

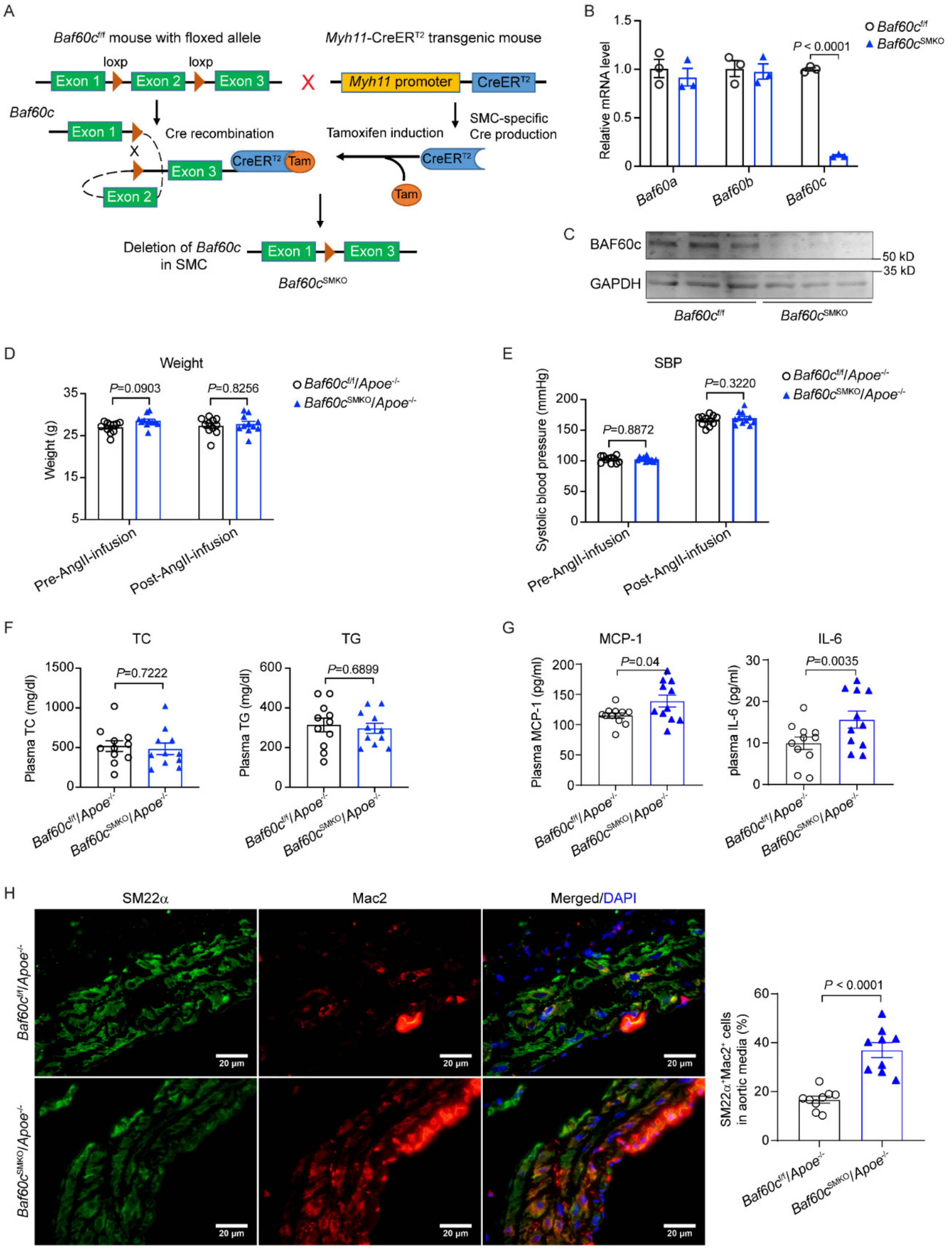

Supplementary Fig. 2. Parameters of the AngII-induced AAA model in *Baf60c*$^{f/f}$/*Apoe*$^{-/-}$ and *Baf60c*^{SMKO}/*Apoe*$^{-/-}$ mice. **A**, Schematics of the gene targeting strategy to generate SMC-specific

Baf60c knockout (*Baf60c*^{SMKO}) mice. **B**, Relative mRNA levels of *Baf60a*, *Baf60b,* and *Baf60c* in the aortas of *Baf60c*$^{f/f}$ and *Baf60c*^{SMKO} mice 9 days after 5 consecutive days of tamoxifen (75 mg/kg/day) intraperitoneal injection. Data are from 3 independent experiments. **C**, Protein abundance of BAF60c in primary aortic SMCs isolated from *Baf60c*$^{f/f}$ and *Baf60c*^{SMKO} mice (n=3/genotype) 9 days after 5 consecutive days of tamoxifen (75 mg/kg/day) intraperitoneal injection. **D**-**H**, Sixteen-week-old male *Baf60c*$^{f/f}$/*Apoe*$^{-/-}$ (n=11) and *Baf60c*^{SMKO}/*Apoe*$^{-/-}$ (n=11) mice were subjected to AngII (1,000 ng/kg/min) infusion with minipumps for 4 weeks. Body weight (**D**) and systolic blood pressure (**E**) before and 4 weeks after AngII minipump implantation. **F**, Plasma total cholesterol (TC) and triglycerides (TG) in AngII-infused *Baf60c*$^{f/f}$/*Apoe*$^{-/-}$ (n=11) and *Baf60c*^{SMKO}/*Apoe*$^{-/-}$ (n=11) mice. **G**, ELISA analysis of MCP-1 and IL-6 in the plasma from AngII-infused *Baf60c*$^{f/f}$/*Apoe*$^{-/-}$ (n=11) and *Baf60c*^{SMKO}/*Apoe*$^{-/-}$ (n=11) mice. **H**, Representative immunofluorescence staining and quantification of SM22α^{+} and Mac2^{+} cells within the aortic wall of suprarenal abdominal aortas from AngII-infused *Baf60c*$^{f/f}$/*Apoe*$^{-/-}$ and *Baf60c*^{SMKO}/*Apoe*$^{-/-}$ mice (n=9/group). Data are presented as mean±SEM. Student's *t*-test for B and F-H. Two-way ANOVA followed by Holm-Sidak post hoc analysis for D-E.

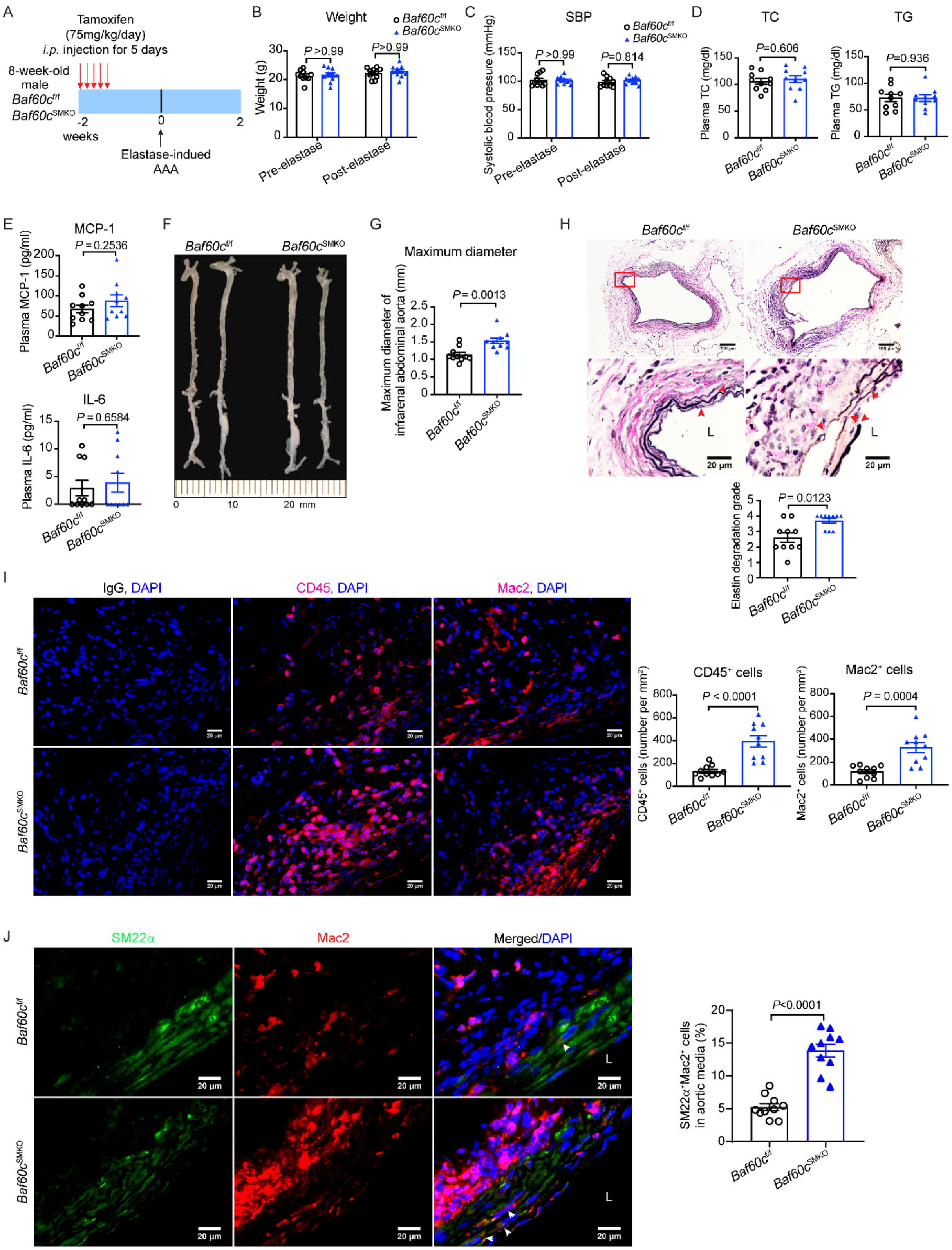
A
Tamoxifen
(75mg/kg/day)
i.p. injection for 5 days
8-week-old male
Baf60c^f/f
Baf60c^SMKO
-2
0
2
weeks
Elastase-indued AAA
B
Weight
Weight (g)
P >0.99
P>0.99
Pre-elastase
Post-elastase
C
SBP
Systolic blood pressure (mmHg)
P >0.99
P=0.814
D
TC
TG
Plasma TC (mg/dl)
Plasma TG (mg/dl)
P=0.606
P=0.936
E
MCP-1
Plasma MCP-1 (pg/ml)
P = 0.2536
IL-6
Plasma IL-6 (pg/ml)
P = 0.6584
F
0
10
20 mm
G
Maximum diameter
Maximum diameter of infrarenal abdominal aorta (mm)
P= 0.0013
H
L
20 μm
Elastin degradation grade
P= 0.0123
I
IgG, DAPI
CD45, DAPI
Mac2, DAPI
CD45⁺ cells
CD45⁺ cells (number per mm²)
P < 0.0001
Mac2⁺ cells
Mac2⁺ cells (number per mm²)
P = 0.0004
J
SM22α
Mac2
Merged/DAPI
SM22α⁺Mac2⁺ cells in aortic media (%)
P<0.0001

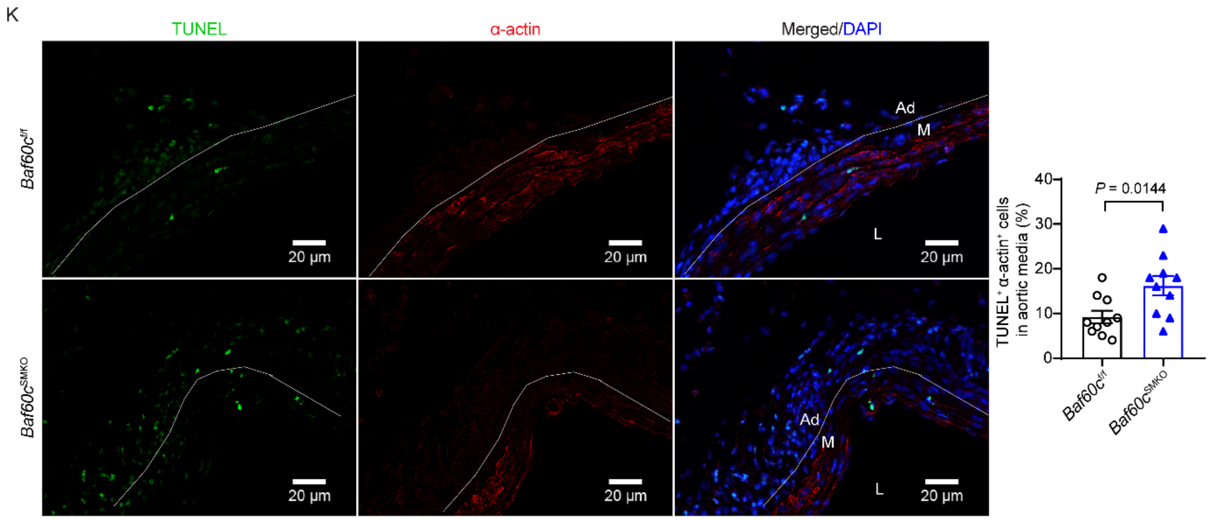

Supplementary Fig. 3. VSMC-BAF60c deficiency aggravates elastase-induced AAA in mice. **A-K**, Ten-week-old male $Baf60c^{f/f}$ (n=10) and $Baf60c^{SMKO}$ (n=10) mice were subjected to an elastase-induced AAA model by treatment of the infrarenal aortas with 30 μl elastase for 30 min. **A**, Schematic diagram of the elastase-induced murine infrarenal AAA model. Body weight (**B**) and systolic blood pressure (**C**) before and 14 days after elastase exposure. Plasma total cholesterol (TC) and triglycerides (TG) (**D**), MCP-1 and IL-6 (**E**) in $Baf60c^{f/f}$ (n=10) and $Baf60c^{SMKO}$ (n=10) 14 days after elastase exposure. **F**, Representative morphology of aortas at the endpoint (14 days after elastase exposure). **G**, Quantification of maximum external diameters of infrarenal abdominal aortas (n=10/group). **H**, Representative Verhoff-Van Gieson staining and quantification analysis of elastin degradation in the infrarenal abdominal aortas from $Baf60c^{f/f}$ and $Baf60c^{SMKO}$ mice (n=10/group). Scale bar=200 μm for the whole aortic sections; scale bar=20μm for the magnified areas. L indicates lumen. **I**, Representative immunofluorescence staining and quantification of leukocyte (CD45$^+$) and macrophage (Mac2$^+$) infiltration in the aortic wall of infrarenal abdominal aortas from $Baf60c^{f/f}$ and $Baf60c^{SMKO}$ mice (n=10/group). Nuclei stained with DAPI are blue. Scale bar=20 μm. **J**, Representative immunofluorescence staining and quantification of SM22α$^+$ and Mac2$^+$ cells within the wall of infrarenal abdominal aortas from $Baf60c^{f/f}$ and $Baf60c^{SMKO}$ mice at the endpoint (n=10/group). **K**,

Representative TUNEL staining (green), SM α-actin (red) immunofluorescent staining and quantification of the apoptotic SM α-actin positive cells in the media of infrarenal abdominal aortas from *Baf60c*$^{f/f}$ and *Baf60c*^{SMKO} mice (n=10/group). Nuclei stained with DAPI are blue. Scale bar=20 μm. Ad indicates adventitia. M indicates media. L indicates lumen. Data are presented as mean±SEM. Two-way ANOVA followed by Holm-Sidak post hoc analysis for B-C. Student's *t*-test for D-E, G, J-K; Mann-Whitney test for H-I.

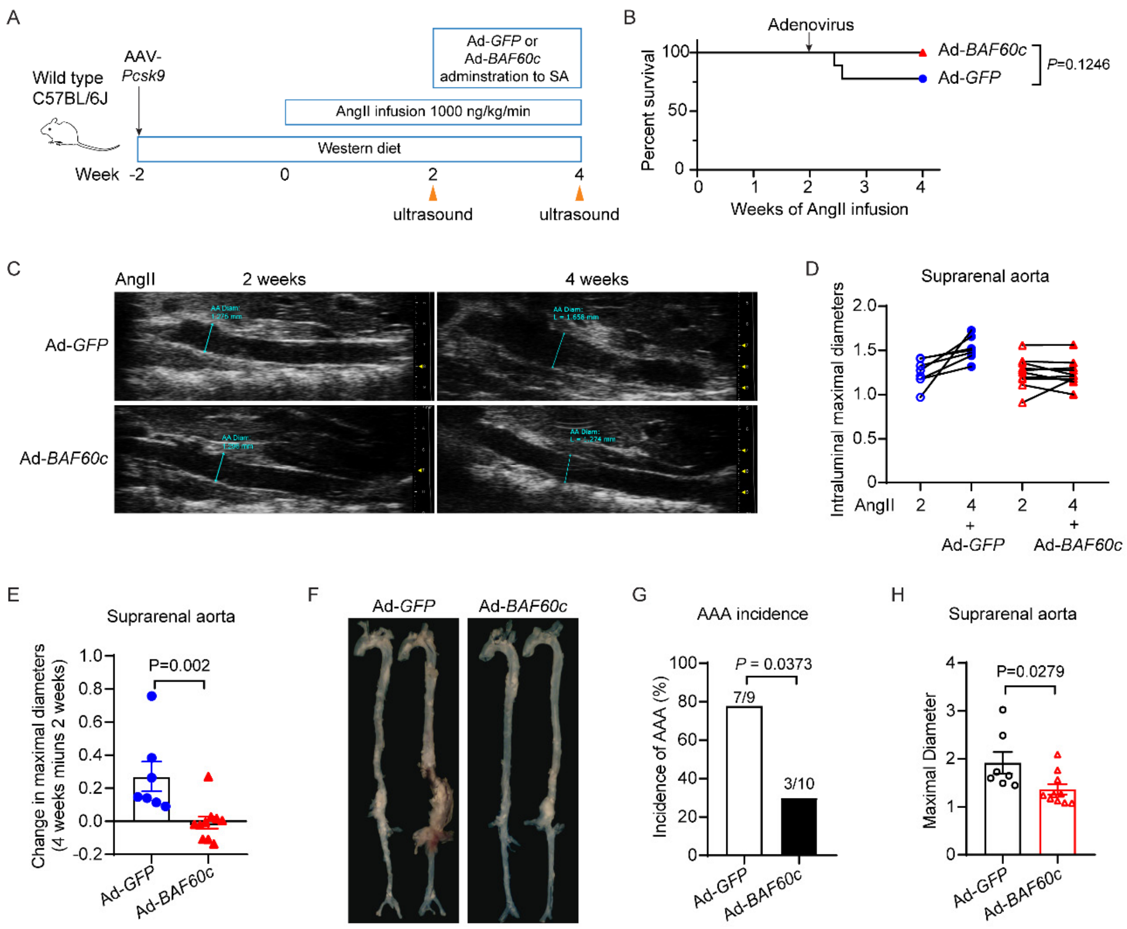

Supplementary Fig. 4. BAF60c inhibits AAA development. A-H, In the Pcsk9/AngII-induced AAA model, after inducing hyperlipidemia by AAV-*Pcsk9.D377Y* and western diet, C57BL/6J mice were infused with AngII (1,000 ng/kg/min) for 4 weeks. Two weeks after AngII infusion, the mice were randomly divided into 2 groups and infected with adenovirus Ad-*BAF60c* (n=10) or Ad-*GFP* (n=9) periadventitially in the suprarenal abdominal aortas. The aortas were assessed by ultrasound at week 2 and week 4 after AngII infusion (**A**). **B**, Kaplan-Meier survival curve of Ad-*GFP* or Ad-*BAF60c* infected mice. **C**, Representative ultrasound images (longitudinally) of the mouse suprarenal abdominal aorta. **D-E**, The change in the internal diameter of the suprarenal abdominal aorta was calculated as [diameter (week 4)] – [diameter (week 2)]. Ad-*GFP*, n=7; Ad-*BAF60c*, n=10. **F**, Representative morphology of aortas at the endpoint. **G**, AAA incidence. **H,** Quantification of maximum external diameters of suprarenal abdominal aortas. n =7 for Ad-*GFP*. n=10 for Ad-*BAF60c*. Data are presented as mean±SEM. Mantel-cox test for B. Chi-square test for G; Mann-Whitney test for E; Student's *t*-test for H.

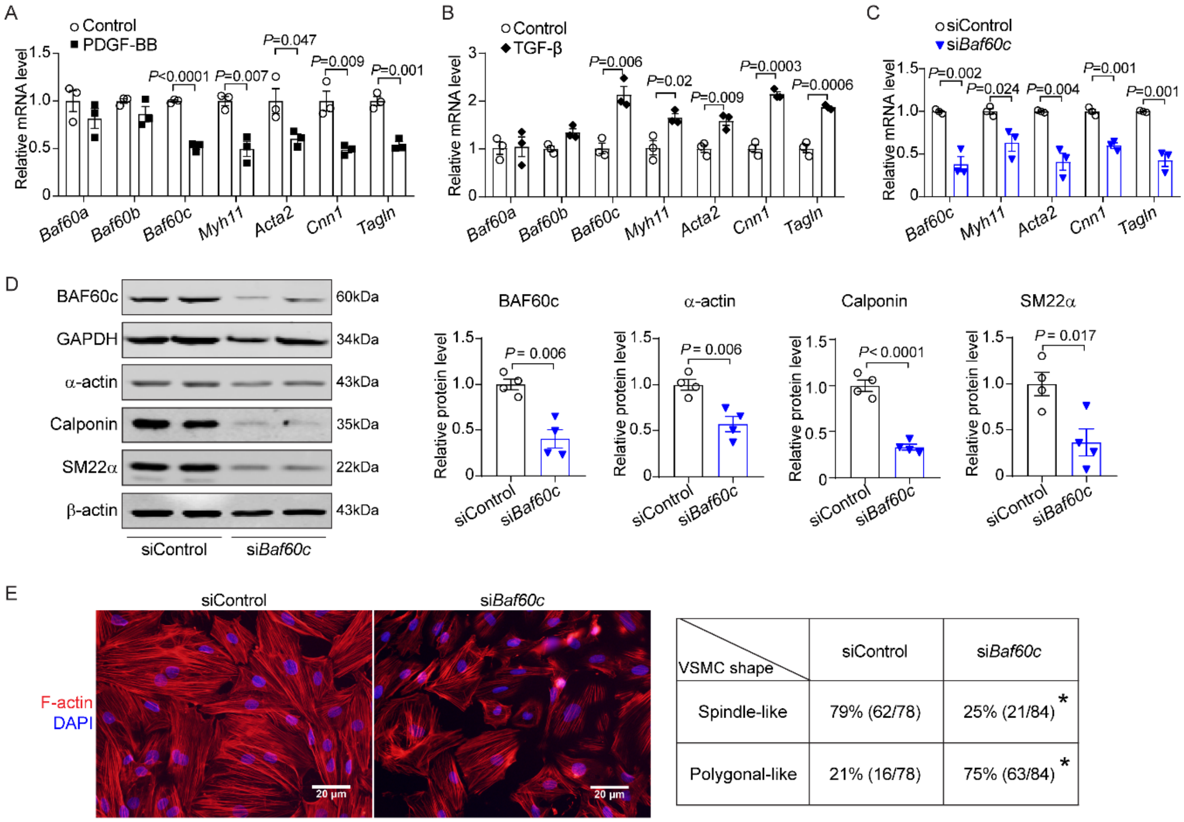

VSMC shape	siControl	siBaf60c
Spindle-like	79% (62/78)	25% (21/84) *
Polygonal-like	21% (16/78)	75% (63/84) *

Supplementary Fig. 5. BAF60c preserves the vascular smooth muscle cell contractile phenotype. **A-B**, A7r5 cells were serum-starved in Opti-MEM for 24h, and then treated with PDGF-BB (**A**, 20 ng/μl) or TGF-β (**B**, 10 ng/ml) for 24h. The mRNA levels of *Baf60a*, *Baf60b*, *Baf60c*, *Myh11*, *Acta2*, *Cnn1,* and *Tagln* were determined by qPCR (n=3 independent experiments). **C-E**, A7r5 cells were transfected with siControl or si*Baf60c* for BAF60c knockdown. After 48h, the cells were serum-starved in Opti-MEM for 24h. **C**, The mRNA levels of *Baf60c*, *Myh11, Acta2*, *Cnn1,* and *Tagln* were determined by qPCR from 3 independent experiments. **D**, Representative Western blot and quantification of the protein abundance of BAF60c, smooth muscle α-actin, calponin, and SM22α. Data are from 4 independent experiments. **E**, Representative immunofluorescence images of F-actin (red) staining in serum-starved A7r5 cells. Nuclei stained with DAPI are blue. Scale bar=20 μm. The right table shows the percentage of spindle-like or polygonal-like cells relative to the total number of cells per group. Data are presented as mean±SEM. Student's *t*-test for A-D. Chi-square test for E with **P*<0.05.

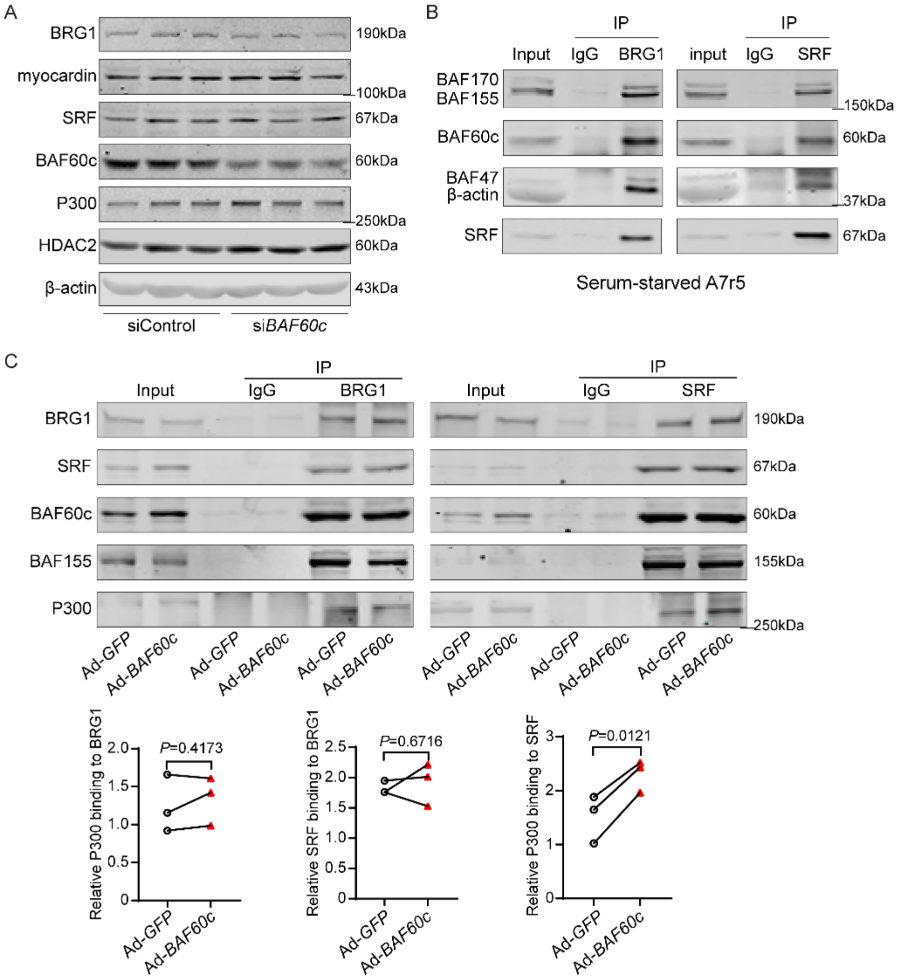

Supplementary Fig. 6. SRF interacts with the SWI/SNF complex. **A**, HASMCs were transfected with siControl or si*BAF60c*. After 48h, the cells were serum-starved in Opti-MEM for 24h. The protein abundance of P300, BRG1, myocardin, SRF, and BAF60c were determined by Western blot. Data are from 3 independent experiments. **B**, A7r5 cells were serum-starved in Opti-MEM for 24h. The nuclear proteins were isolated and subjected to CoIP assays using antibodies against BRG1 or SRF, with IgG as the negative control. Three independent experiments were performed. **C**, HASMCs were infected with Ad-*GFP* or Ad-*BAF60c* (10 MOI). After 48h, the cells were serum-starved in Opti-MEM for 24h and the nuclear proteins were isolated and subjected to CoIP assays using antibodies against BRG1 or SRF. IgG was used as the negative control. Three independent experiments were performed. Data are presented as mean±SEM. Paired *t*-test for C.

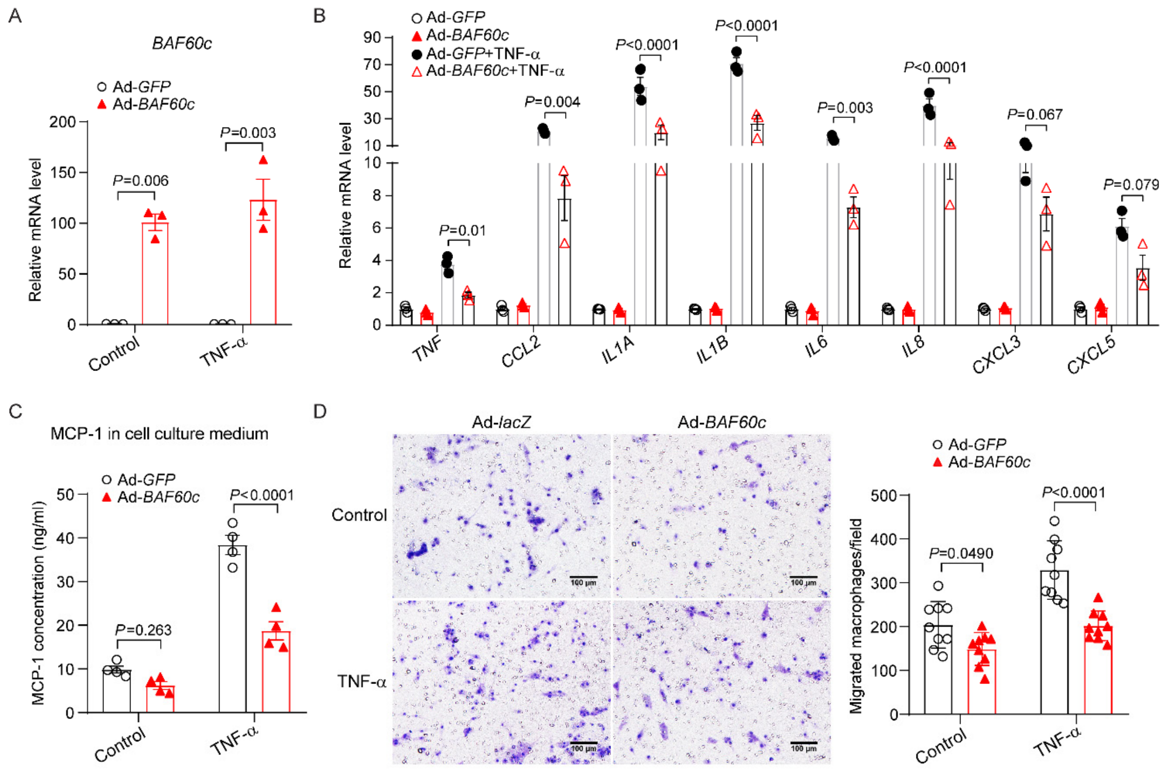

Supplementary Fig. 7. BAF60c inhibits VSMC inflammation. **A-B**, HASMCs were infected with Ad-*GFP*, Ad-*BAF60c* (10 MOI). After 48h, the cells were serum-starved in Opti-MEM for 24h and then treated with TNF-α (20 ng/ml) or vehicle (Control) for 24h. Total RNA was extracted for qPCR to assess the expression of *BAF60c* and inflammation-related genes. Data are from 3 independent experiments. **C**, The MCP-1 concentration in the cell culture medium of HASMC infected with Ad-*GFP* or Ad-*BAF60c* (10 MOI), followed by stimulation with or without TNF-α (20 ng/ml, 24h) was measured by ELISA. Data are from 4 independent experiments. **D**, Representative images (magnified field, left) and quantitative analysis (right) of the bone marrow-derived macrophages (isolated from wildtype mice) in the transwell migration assay when co-cultured with HASMC infected with Ad-*GFP* or Ad-*BAF60c* (10 MOI), and treated with TNF-α (20 ng/ml) stimulation or vehicle control in the lower well before the co-culture (n=9 images/group). Data are presented as mean±SEM. Two-way ANOVA followed by Holm-Sidak post hoc analysis for A-D.

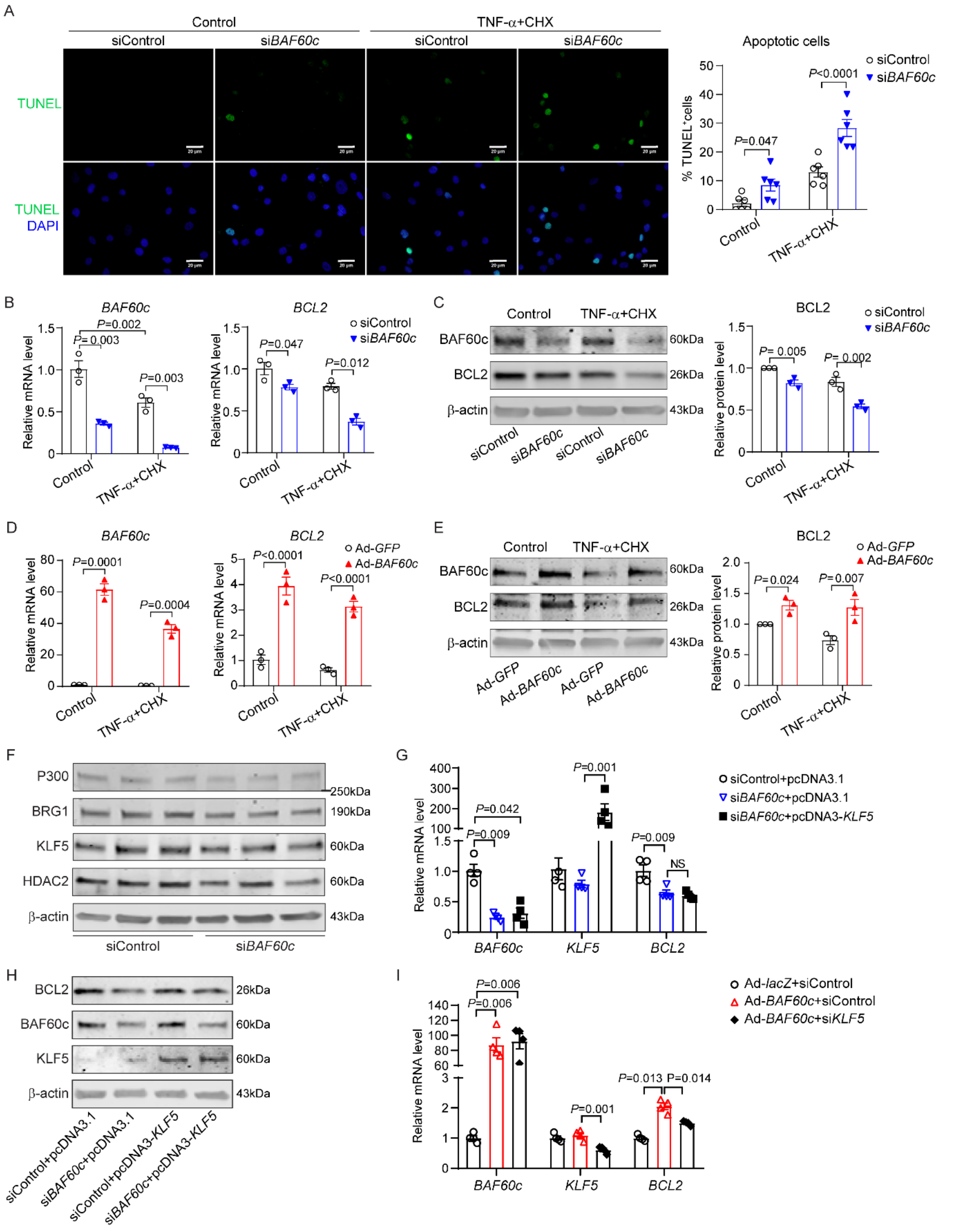

Supplementary Fig. 8. BAF60c-dependent increase in BCL2 requires KLF5. **A**, Representative images of TUNEL staining (green) and quantification of apoptotic HASMC transfected with

siControl or si*BAF60c* (30 nM), and subsequently stimulated for 6h with TNF-α (100ng/ml) and cycloheximide (CHX, 20 μM) 48h after siRNA transfection. **B**-**E**, HASMCs were transfected with siControl, si*BAF60c* (30 nM) (B-C) or infected with Ad-*GFP*, Ad-*BAF60c* (10 MOI) (D-E). After 48h, the cells were stimulated with TNF-α (100ng/ml) and CHX (20 μM) for 6h. The expression of BAF60c and BCL2 was assessed by qPCR (B and D) and Western blot (C and E) from 3 independent experiments. **F**, HASMCs were transfected with siControl or si*BAF60c* (30nM). After 48h, the cells were serum-starved in Opti-MEM for 24h, and the protein abundance of P300, HDAC2, BRG1, and KLF5, relative to β-actin were determined by Western blot. Data are from 3 independent experiments. **G**-**H**, HASMCs were transfected with siControl or si*BAF60c* (30 nM), and pcDNA3.1 or pcDNA3-*KLF5*. After 48h, the cells were serum-starved in Opti-MEM for 24h. qPCR (B) and Western blot (C) were used to determine the expression of BAF60c, KLF5, and BCL2. **I**, HASMCs were transfected with siControl or si*KLF5* (30nM), and infected with Ad-*lacZ*, Ad-*BAF60c* (10 MOI). After 48h, the cells were serum-starved in Opti-MEM for 24h. qPCR was used to determine the expression of *BAF60c*, *KLF5,* and *BCL2* expression. Data are presented as mean±SEM. Two-way ANOVA followed by Holm-Sidak post hoc analysis for A-E, G, and I.

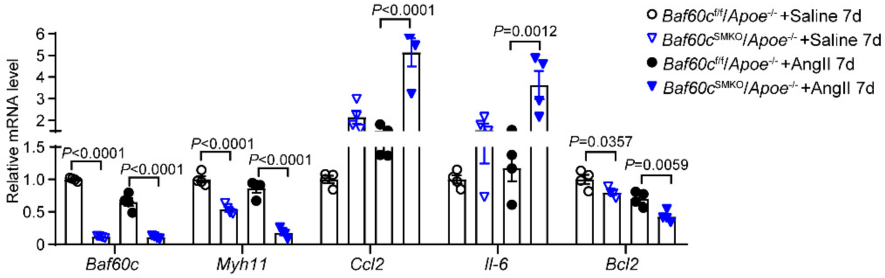

Supplementary Fig. 9. Twelve-week-old male *Baf60c*$^{f/f}$/*Apoe*$^{-/-}$ and *Baf60c*^{SMKO}/*Apoe*$^{-/-}$ mice (n=4/group) were subjected to saline or AngII (1,000 ng/kg/min) infusion with minipumps for 7 days. The mRNA levels of *Baf60c*, *Myh11*, *Ccl2*, *Il-6,* and *Bcl2*, relative to β-actin, were determined by qPCR in mouse suprarenal abdominal aortas. Data are presented as mean±SEM. Two-way ANOVA followed by Holm-Sidak post hoc analysis.
